# Supplementary figures and images for: Functional diversification gave rise to allelic specialization in a rice NLR immune receptor pair
Source: eLife. 2021 Nov 16;10:e71662. doi: 10.7554/eLife.71662 (PMC8631799; doi:10.7554/eLife.71662)

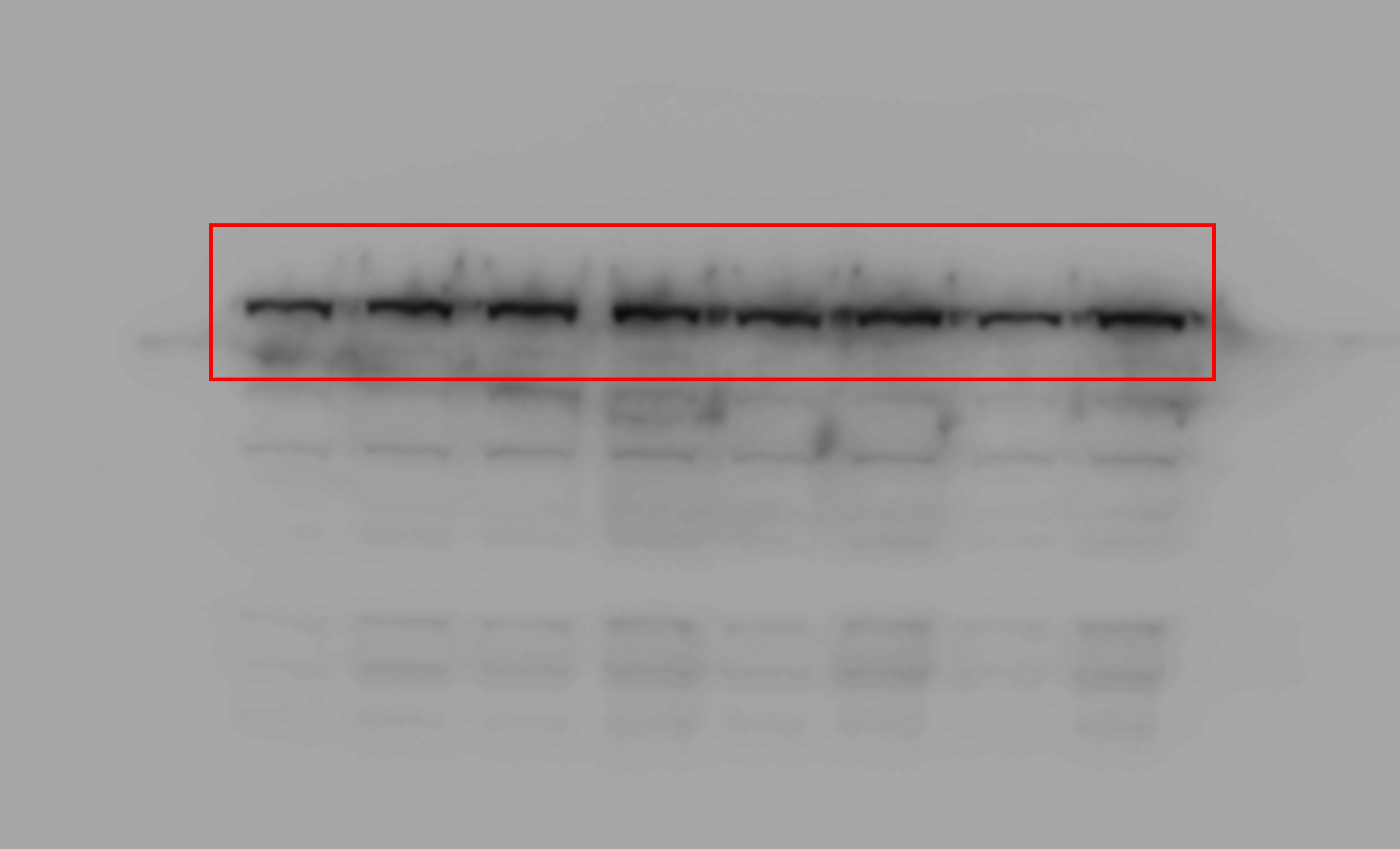

Supplement: Source data 1. [file elife-71662-supp1.zip › Figure 1ΓÇôfigure supplement 3ΓÇôsource data 1.tif]

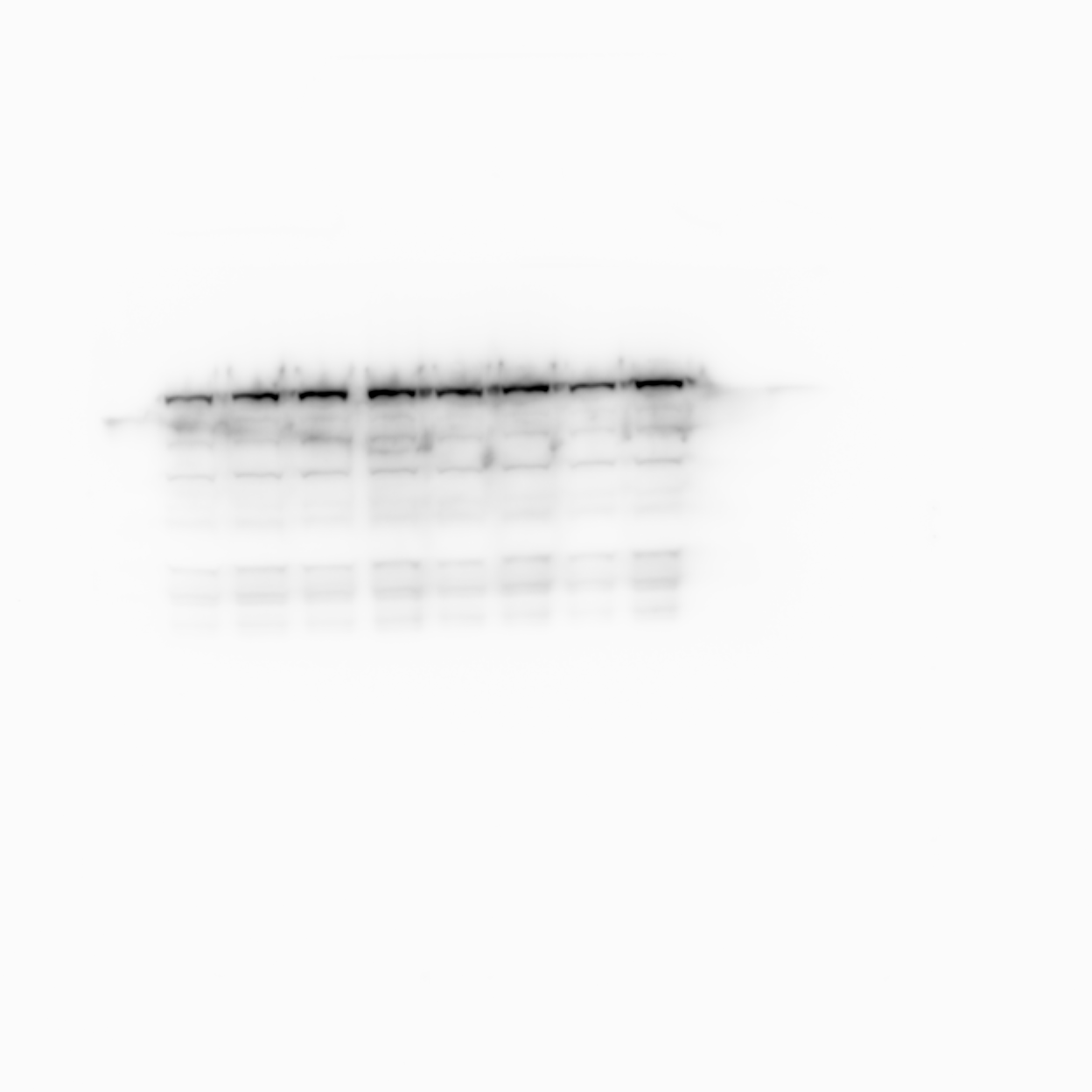

Supplement: Source data 1. [file elife-71662-supp1.zip › Figure 1ΓÇôfigure supplement 3ΓÇôsource data 2.tif]

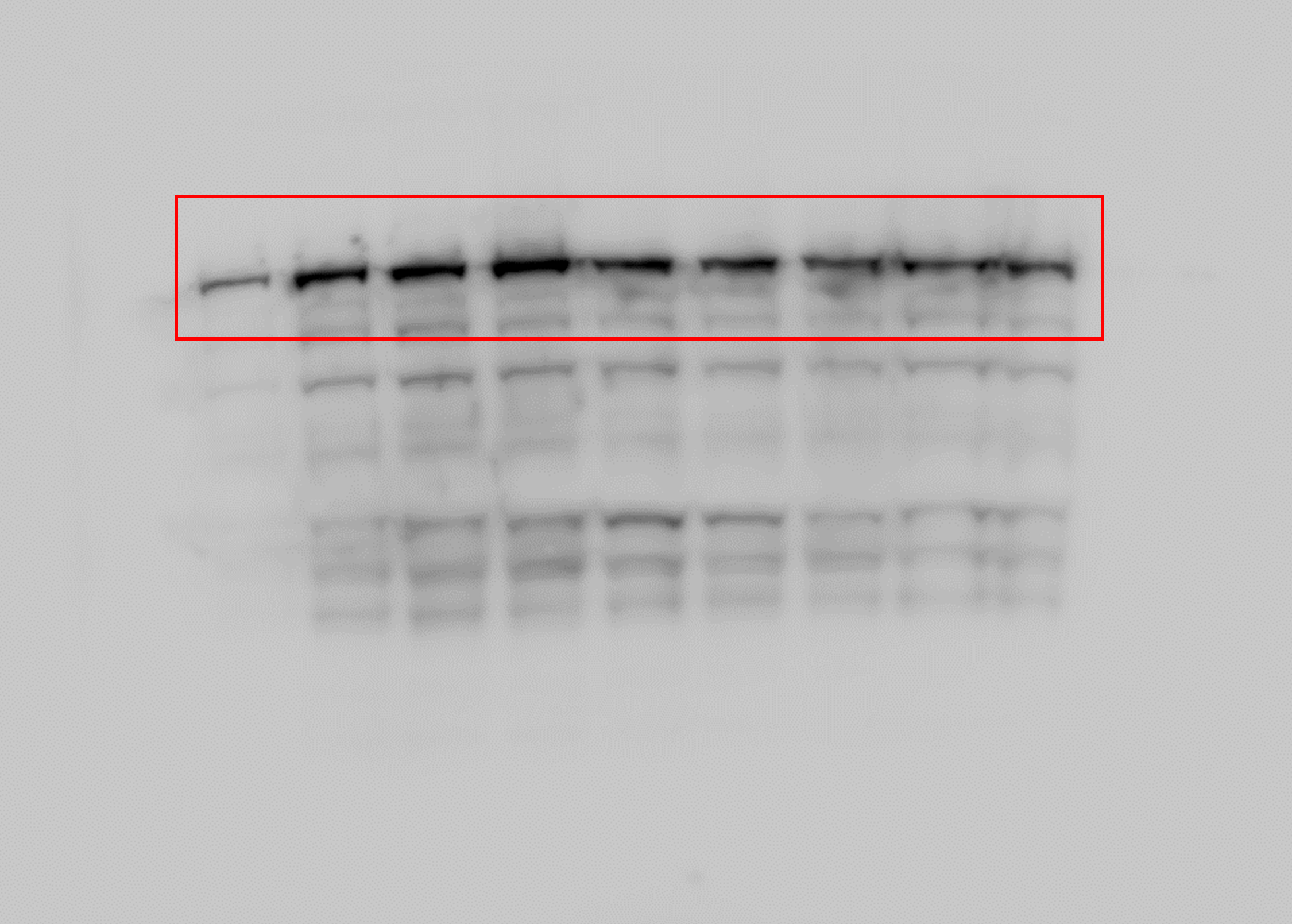

Supplement: Source data 1. [file elife-71662-supp1.zip › Figure 5ΓÇôfigure supplement 4ΓÇôsource data 1.tif]

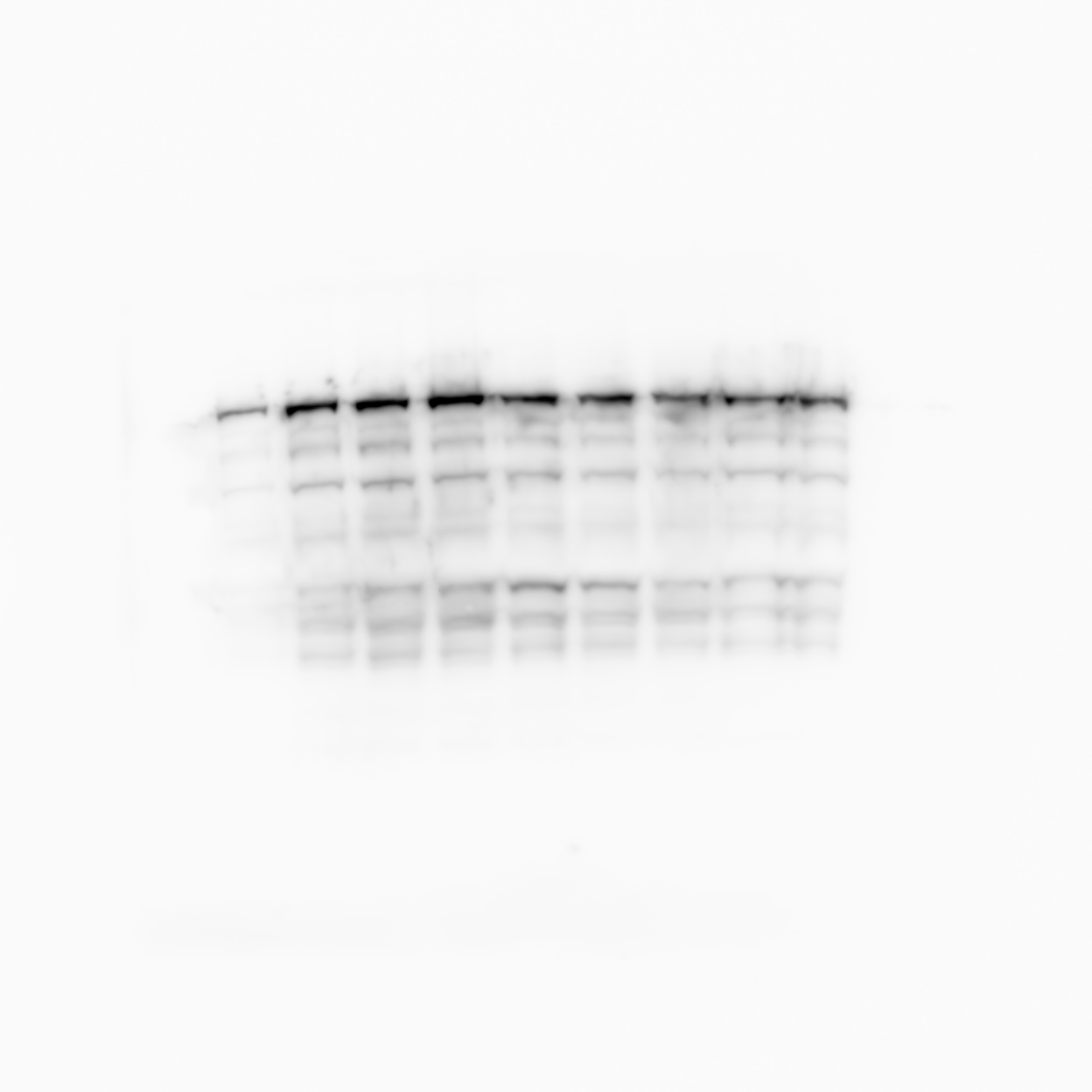

Supplement: Source data 1. [file elife-71662-supp1.zip › Figure 5ΓÇôfigure supplement 4ΓÇôsource data 2.tif]

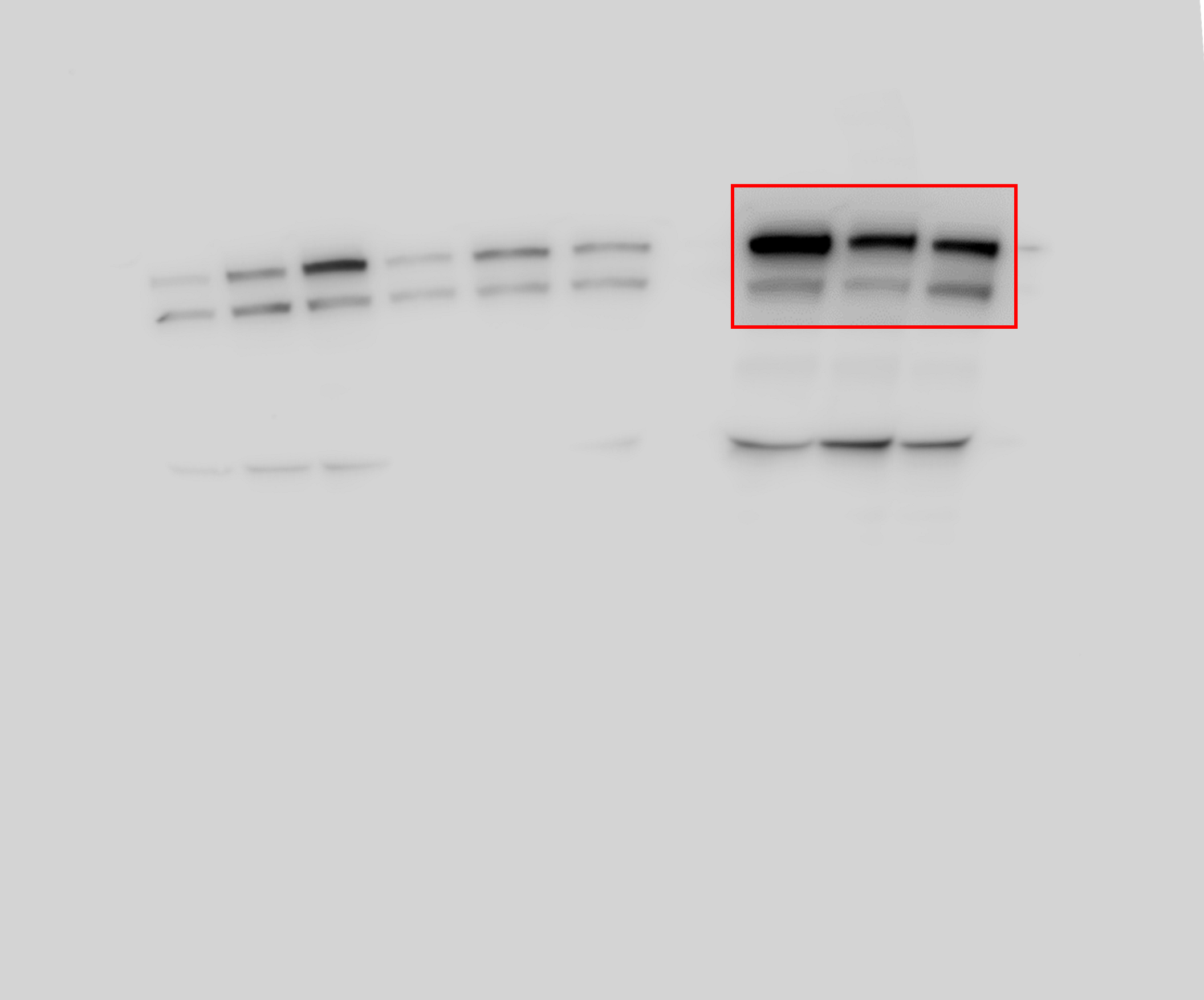

Supplement: Source data 1. [file elife-71662-supp1.zip › Figure 5ΓÇôfigure supplement 4ΓÇôsource data 3.tif]

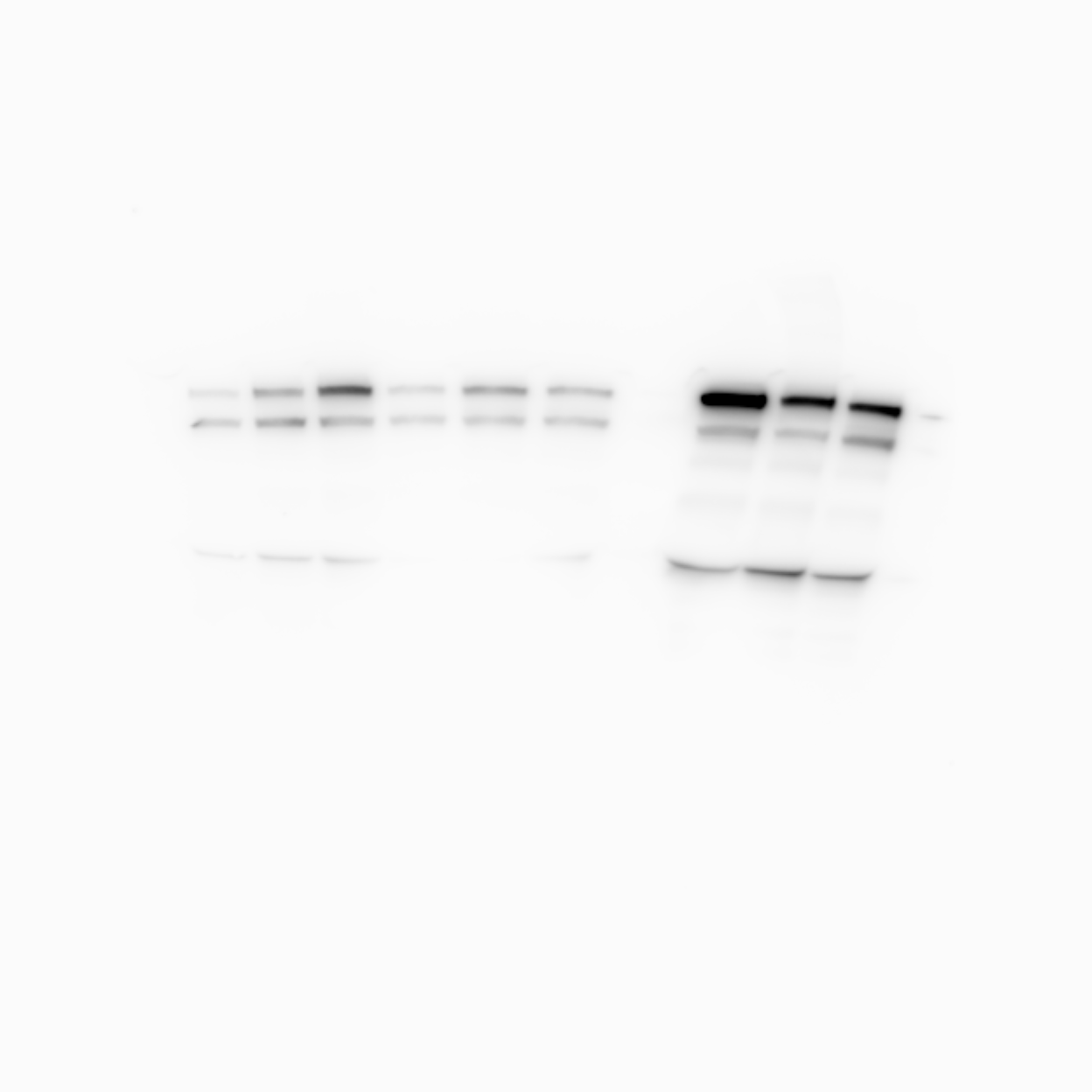

Supplement: Source data 1. [file elife-71662-supp1.zip › Figure 5ΓÇôfigure supplement 4ΓÇôsource data 4.tif]

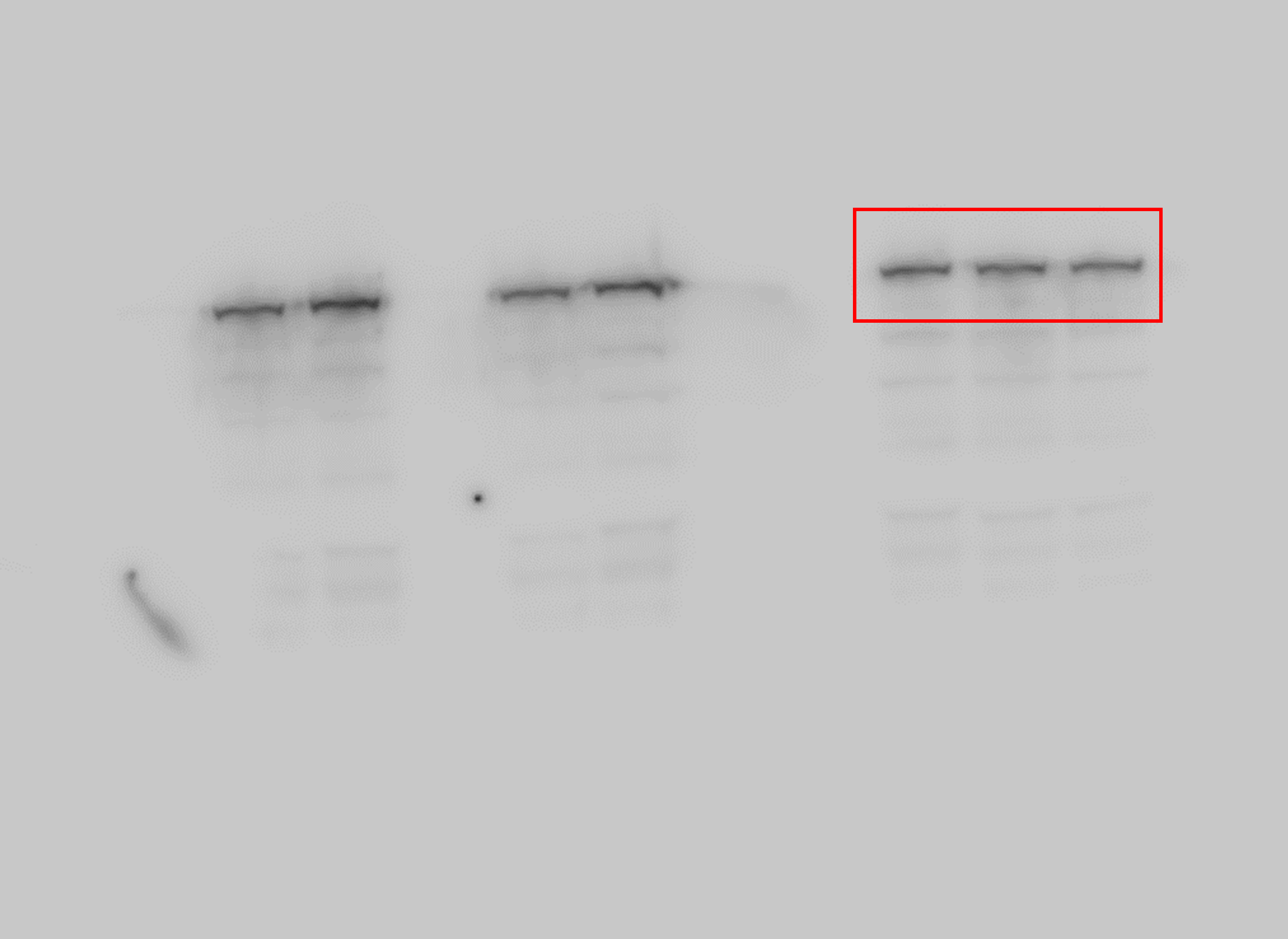

Supplement: Source data 1. [file elife-71662-supp1.zip › Figure 6ΓÇôfigure supplement 2ΓÇôsource data 1.tif]

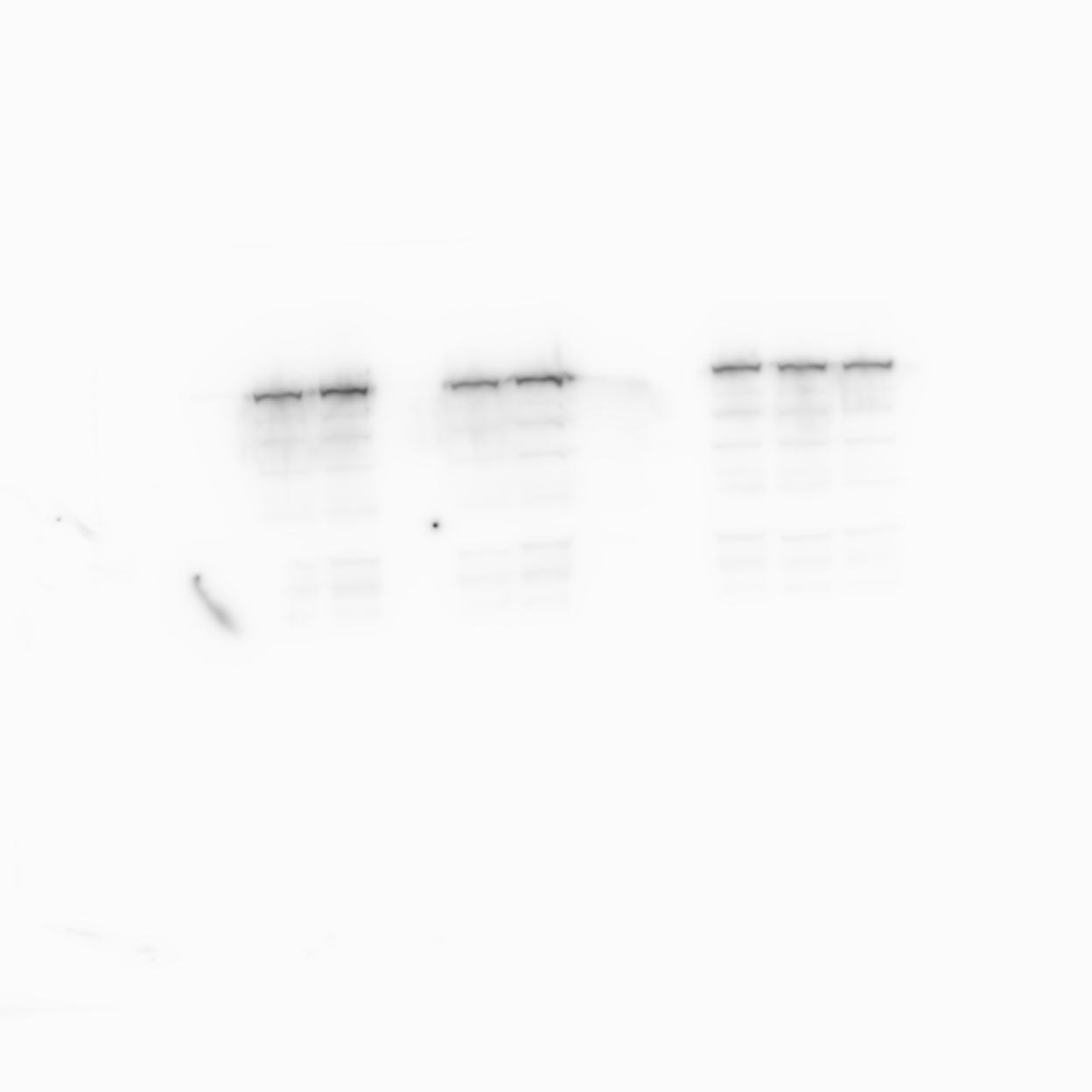

Supplement: Source data 1. [file elife-71662-supp1.zip › Figure 6ΓÇôfigure supplement 2ΓÇôsource data 2.tif]

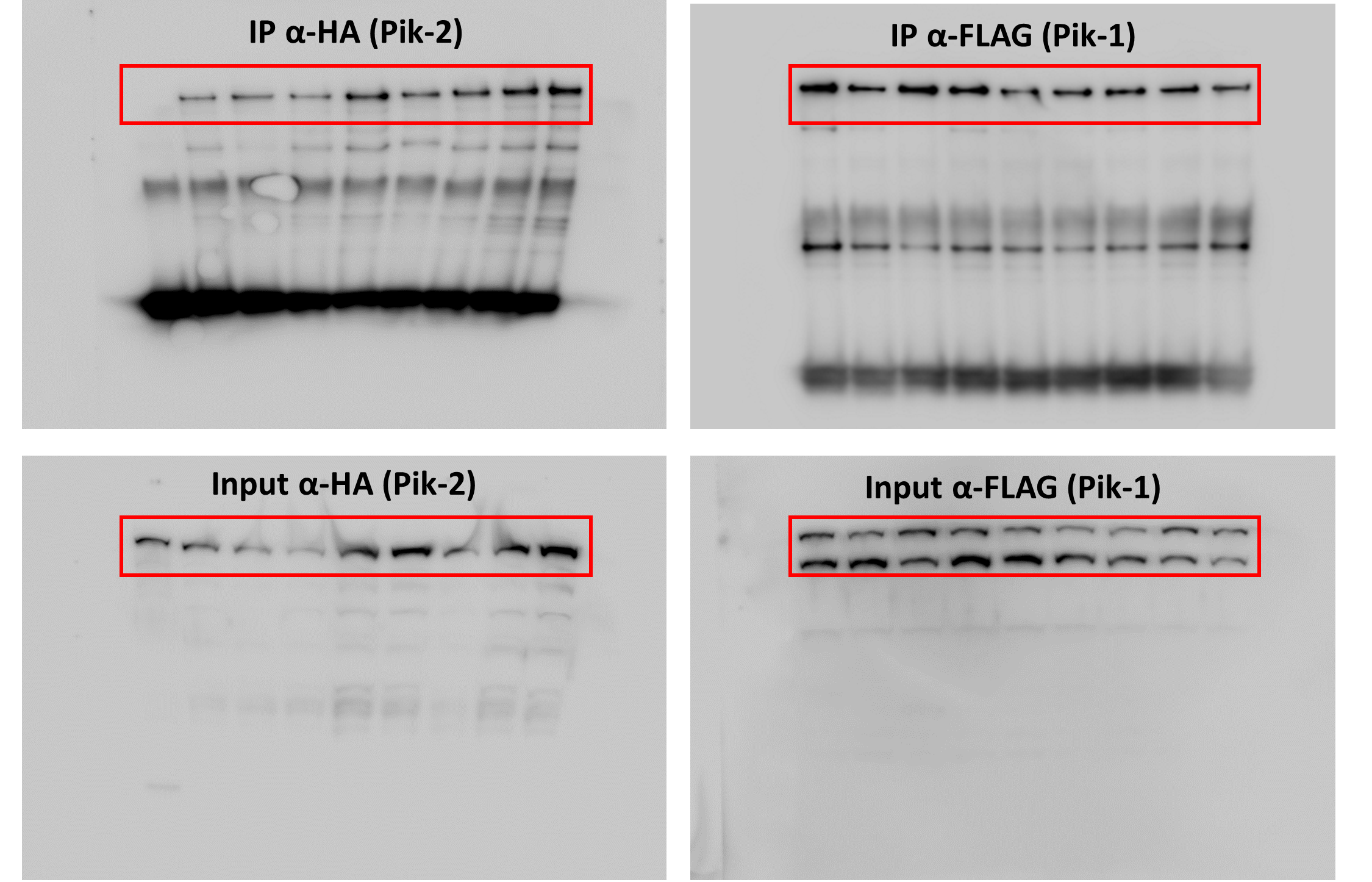

Supplement: Source data 1. [file elife-71662-supp1.zip › Figure 7ΓÇôfigure supplement 1ΓÇôsource data 1.tif]

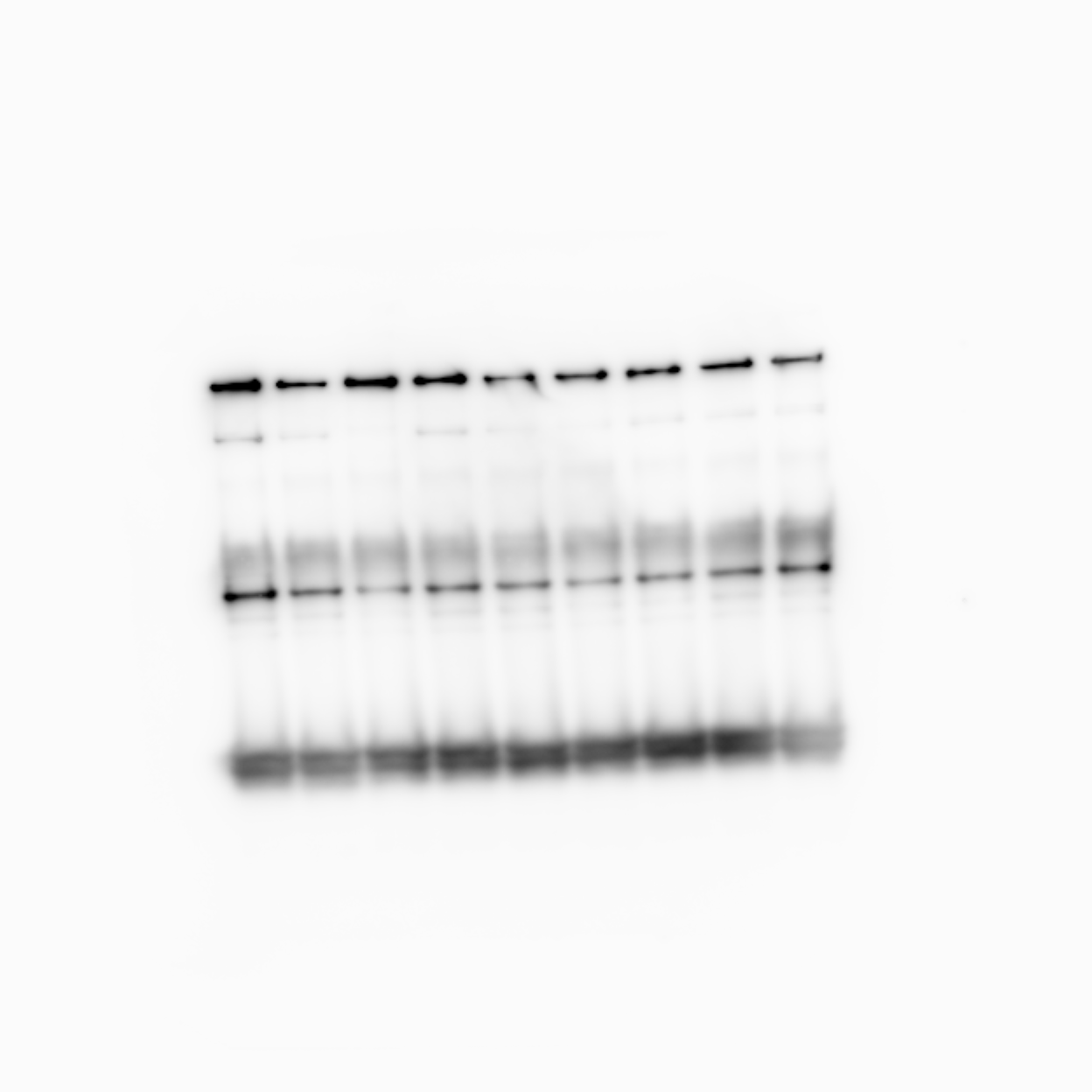

Supplement: Source data 1. [file elife-71662-supp1.zip › Figure 7ΓÇôfigure supplement 1ΓÇôsource data 2.tif]

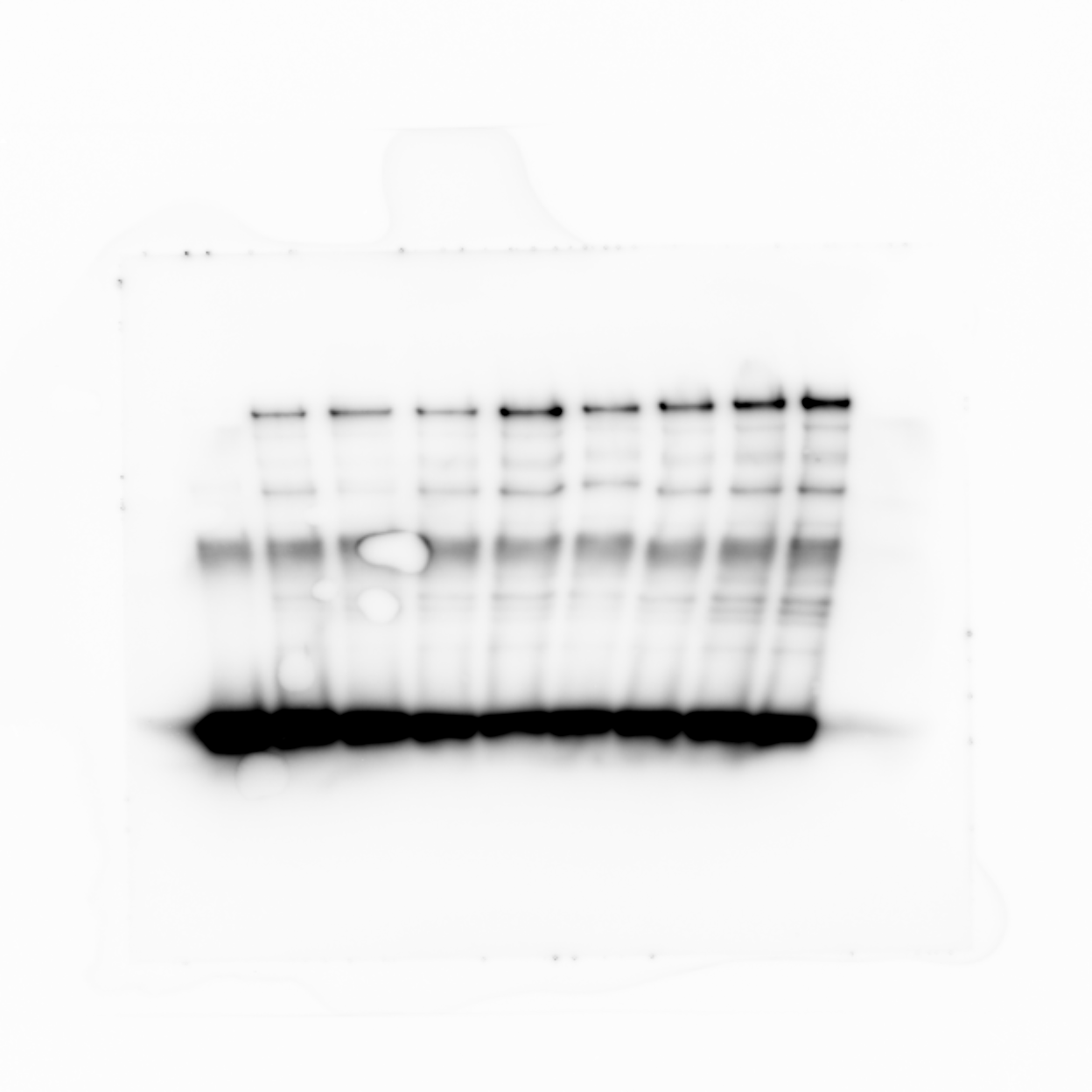

Supplement: Source data 1. [file elife-71662-supp1.zip › Figure 7ΓÇôfigure supplement 1ΓÇôsource data 3.tif]

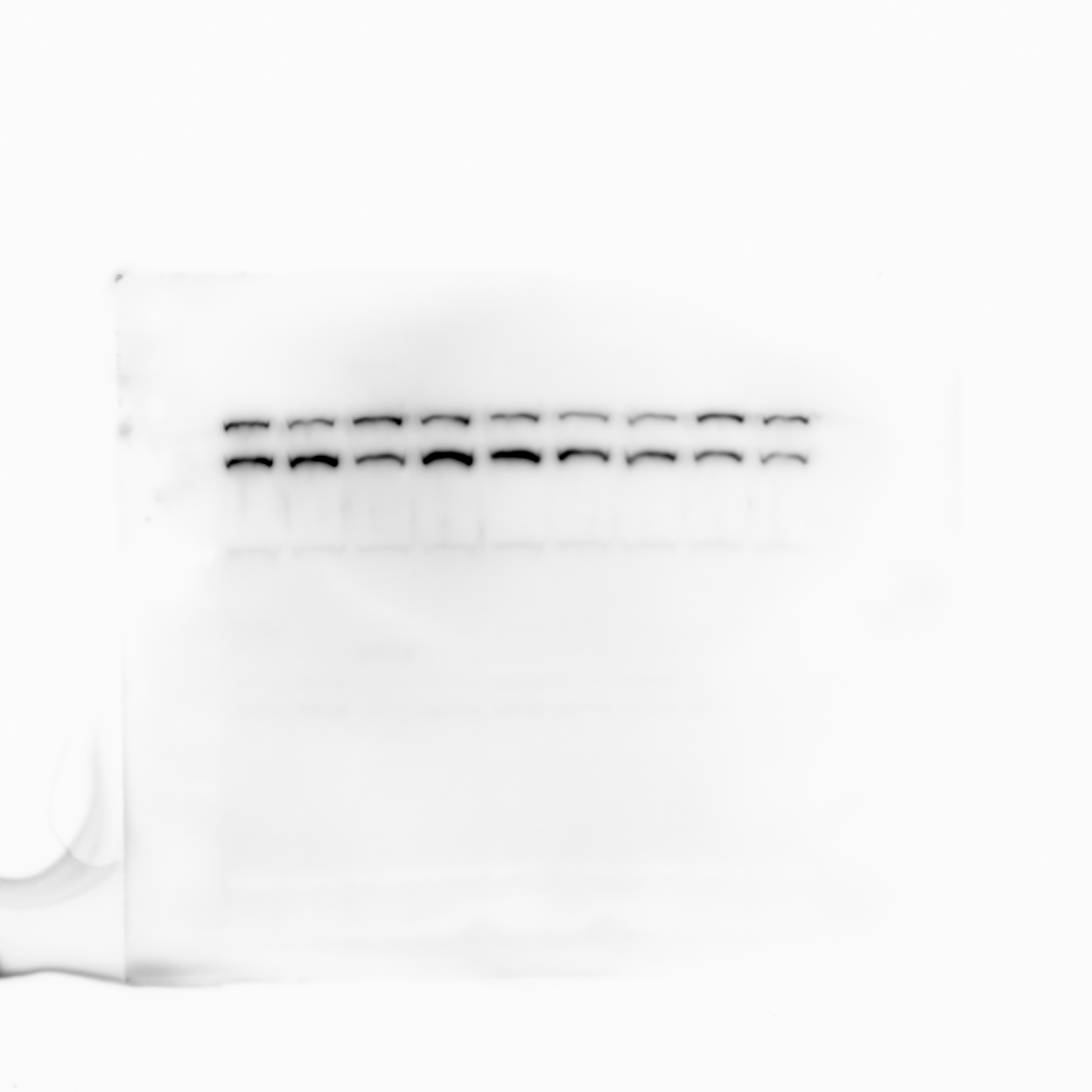

Supplement: Source data 1. [file elife-71662-supp1.zip › Figure 7ΓÇôfigure supplement 1ΓÇôsource data 4.tif]

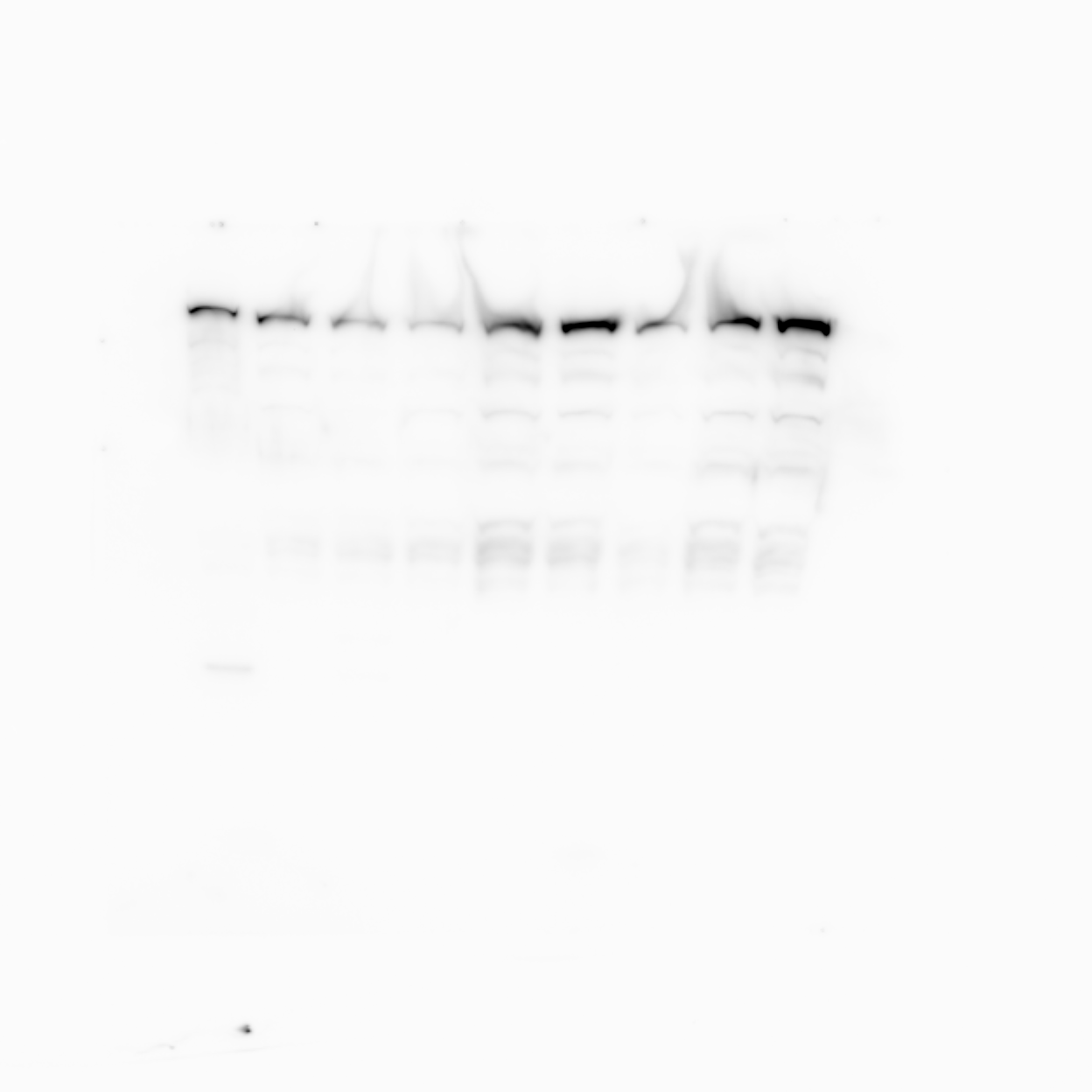

Supplement: Source data 1. [file elife-71662-supp1.zip › Figure 7ΓÇôfigure supplement 1ΓÇôsource data 5.tif]

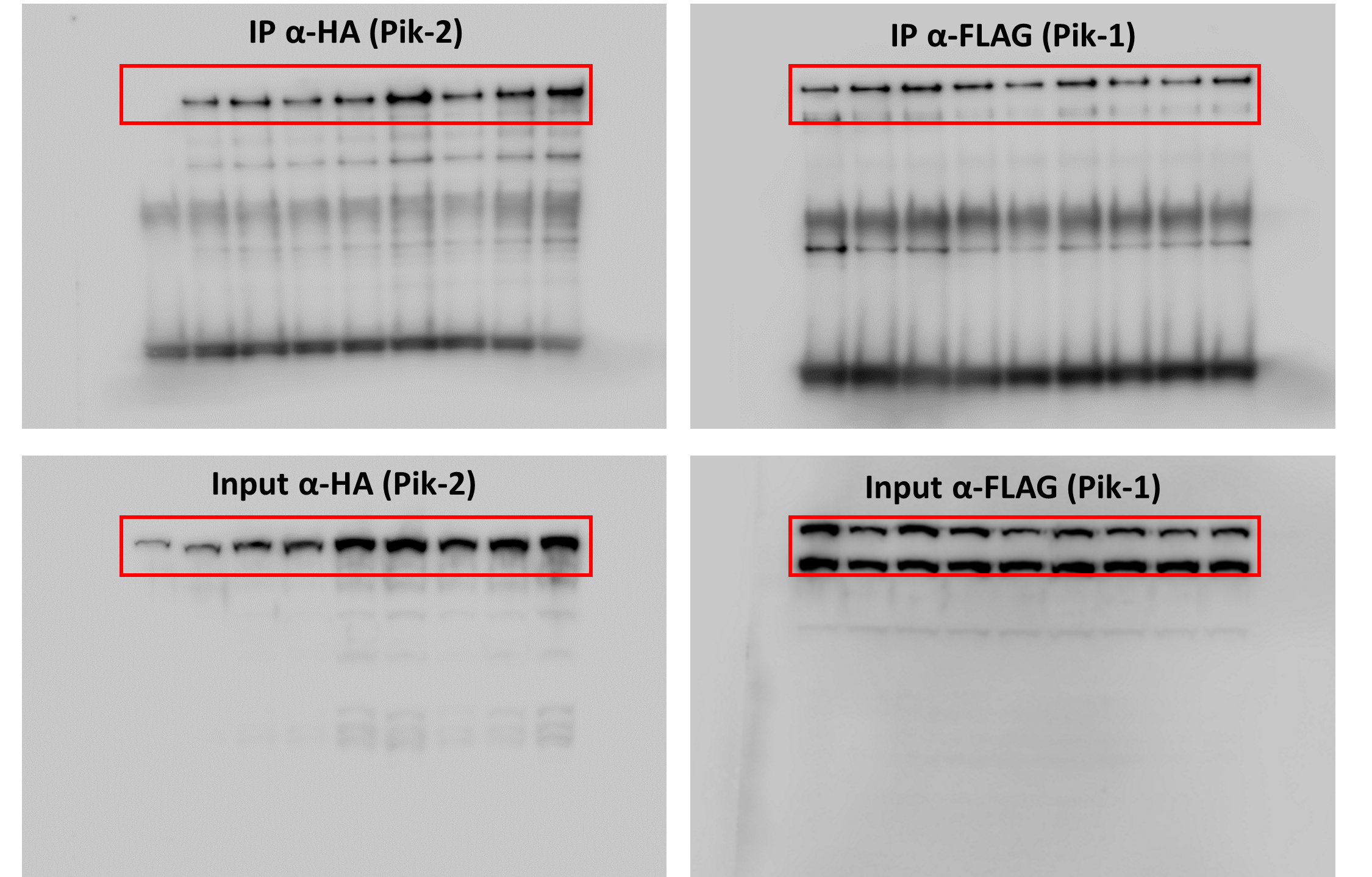

Supplement: Source data 1. [file elife-71662-supp1.zip › Figure 7ΓÇôfigure supplement 1ΓÇôsource data 6.tif]

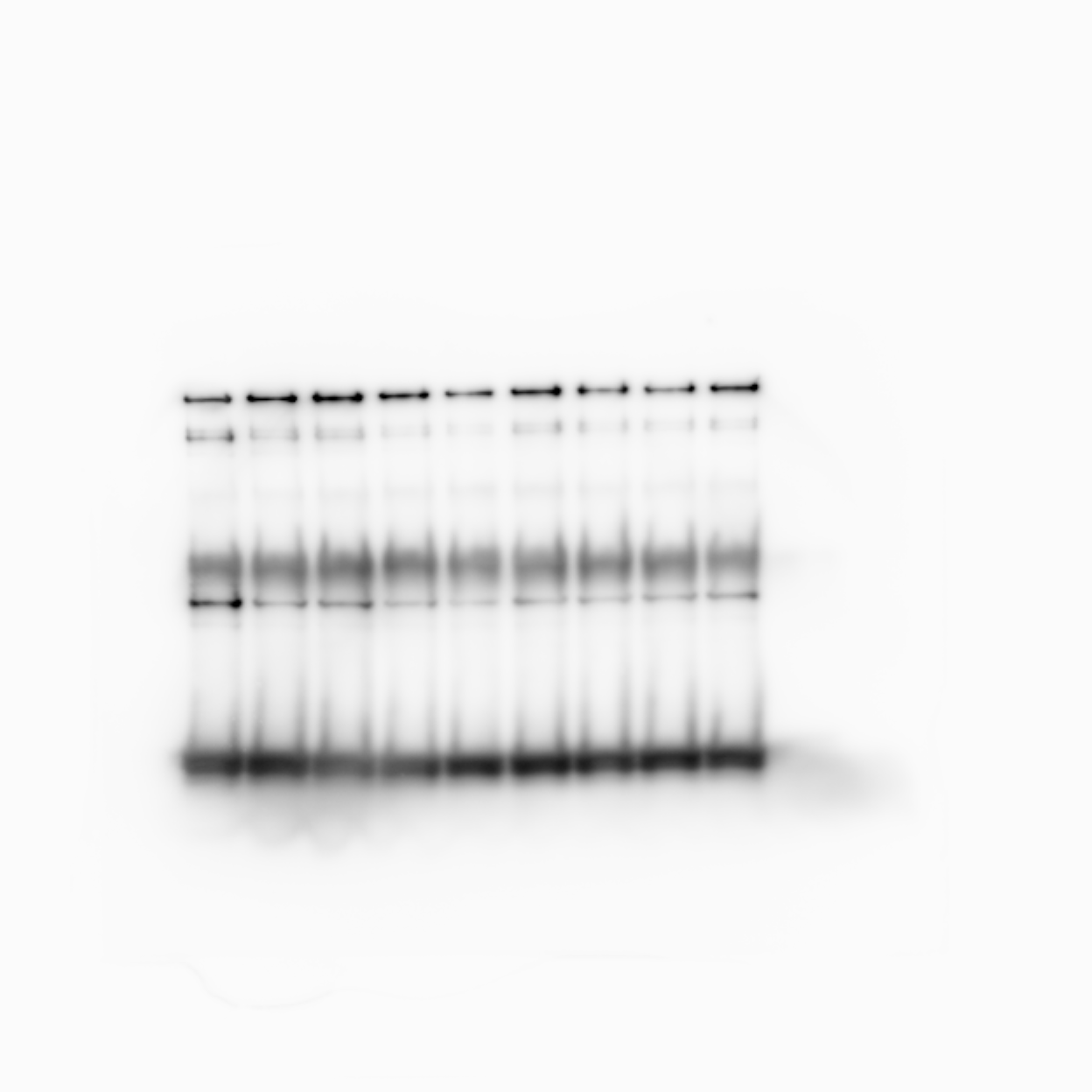

Supplement: Source data 1. [file elife-71662-supp1.zip › Figure 7ΓÇôfigure supplement 1ΓÇôsource data 7.tif]

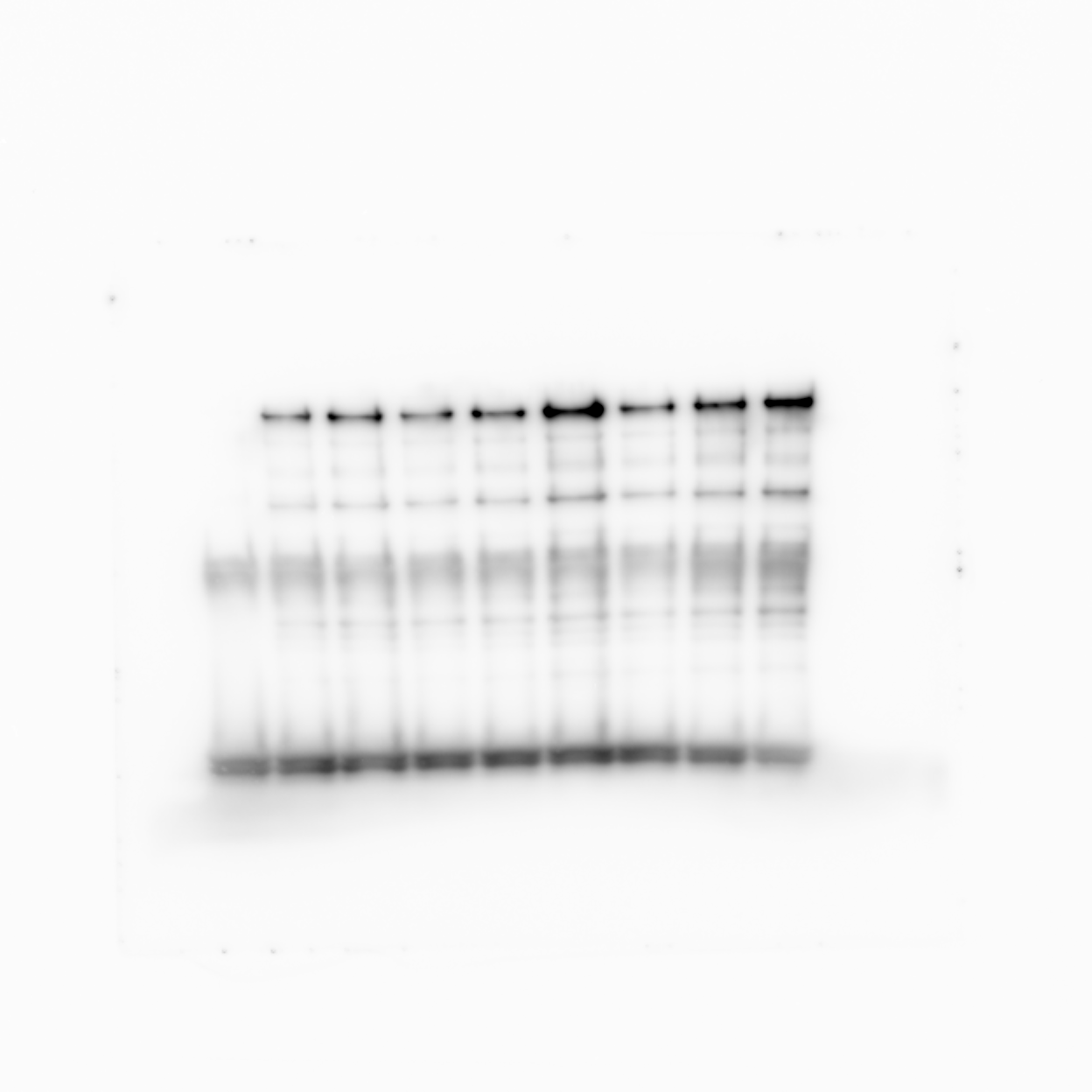

Supplement: Source data 1. [file elife-71662-supp1.zip › Figure 7ΓÇôfigure supplement 1ΓÇôsource data 8.tif]

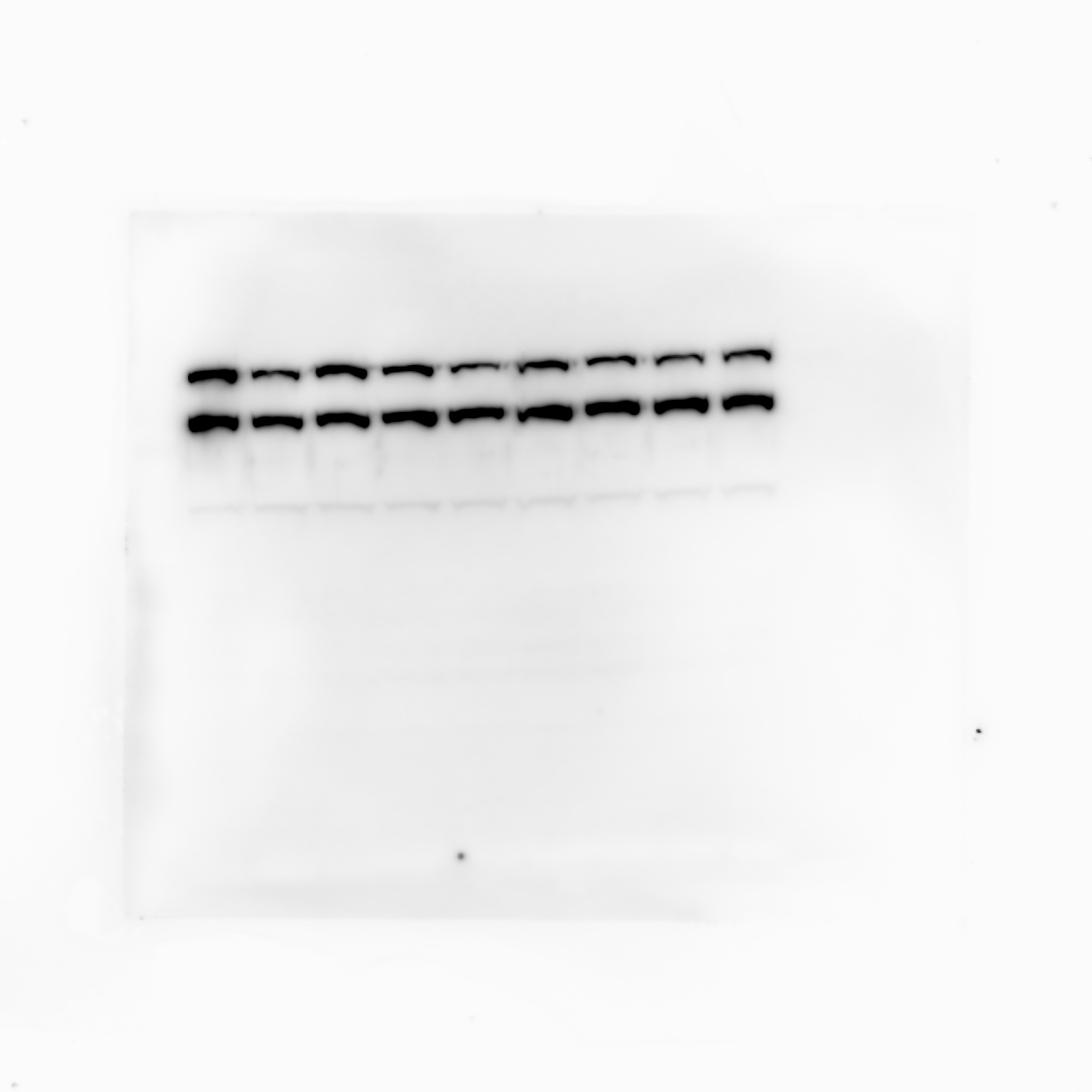

Supplement: Source data 1. [file elife-71662-supp1.zip › Figure 7ΓÇôfigure supplement 1ΓÇôsource data 9.tif]

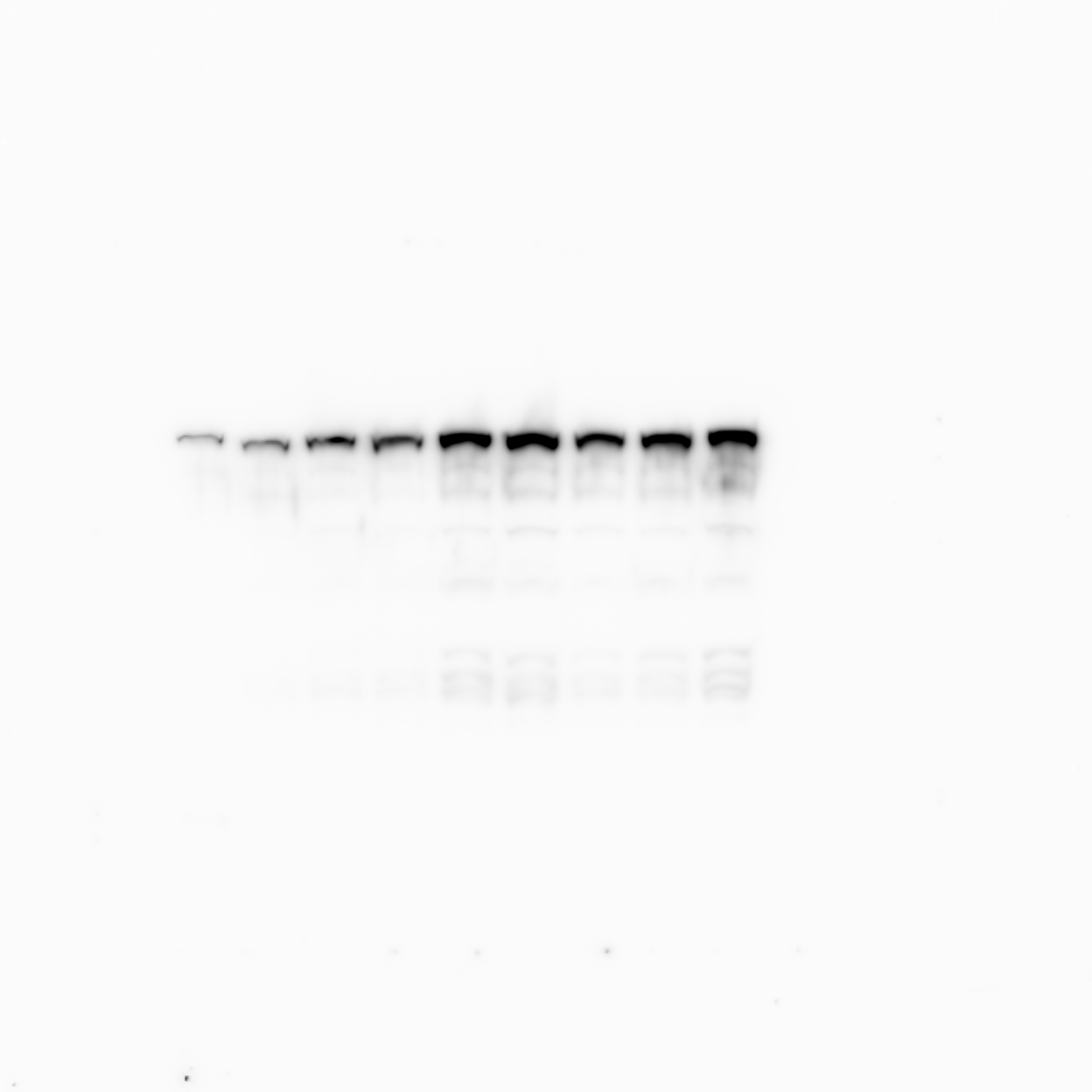

Supplement: Source data 1. [file elife-71662-supp1.zip › Figure 7ΓÇôfigure supplement 1ΓÇôsource data 10.tif]

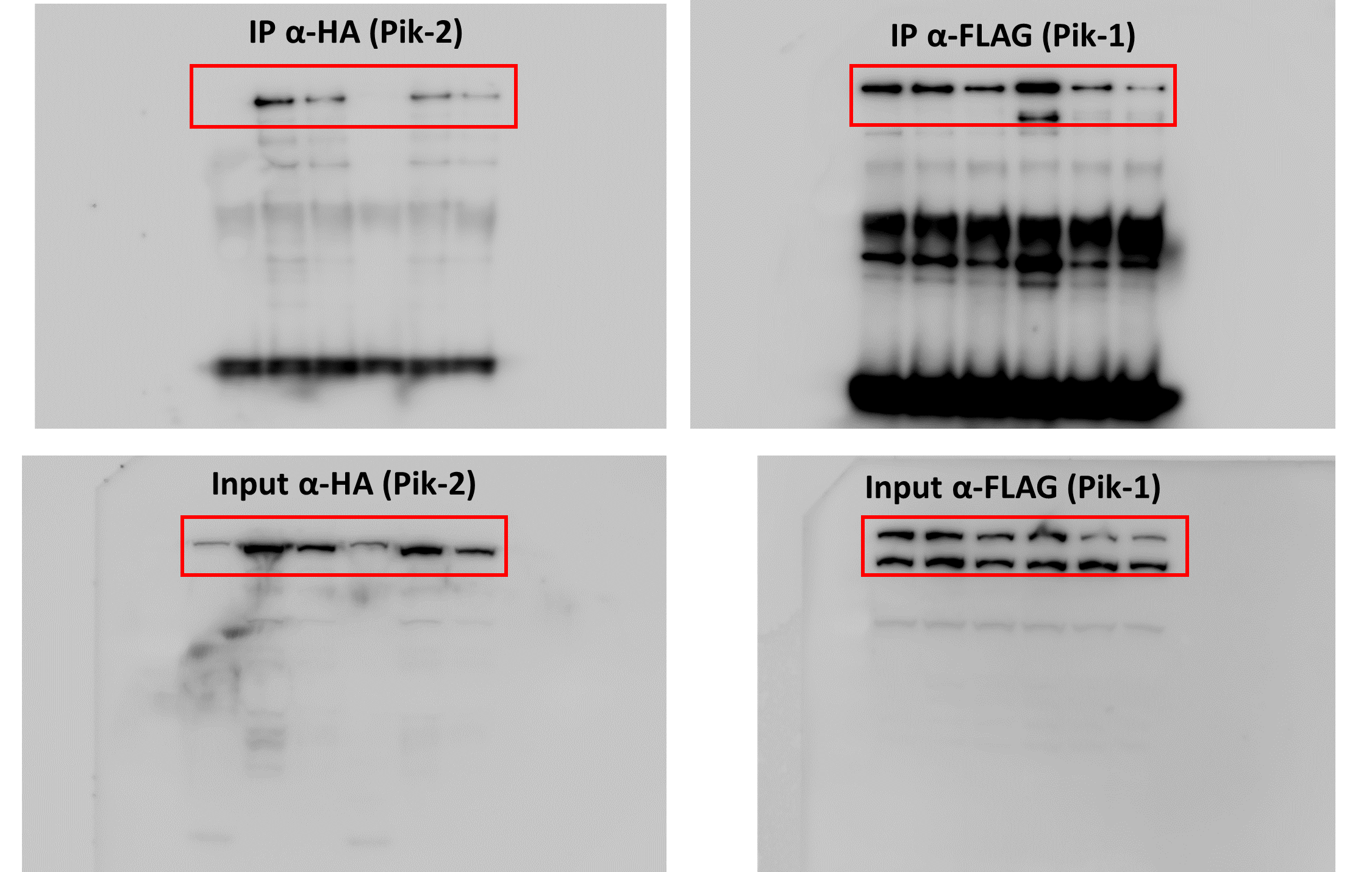

Supplement: Source data 1. [file elife-71662-supp1.zip › Figure 7ΓÇôfigure supplement 2ΓÇôsource data 1.tif]

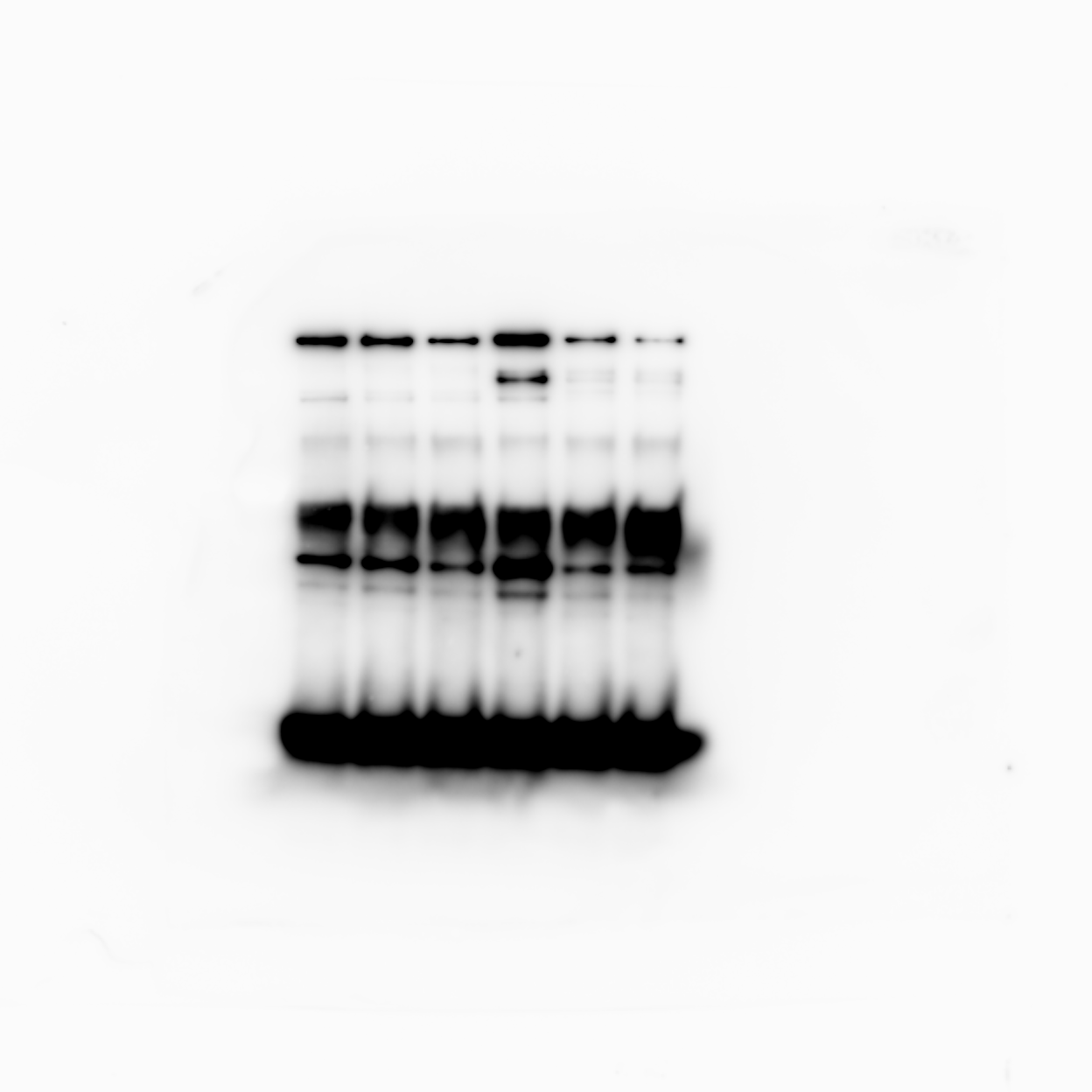

Supplement: Source data 1. [file elife-71662-supp1.zip › Figure 7ΓÇôfigure supplement 2ΓÇôsource data 2.tif]

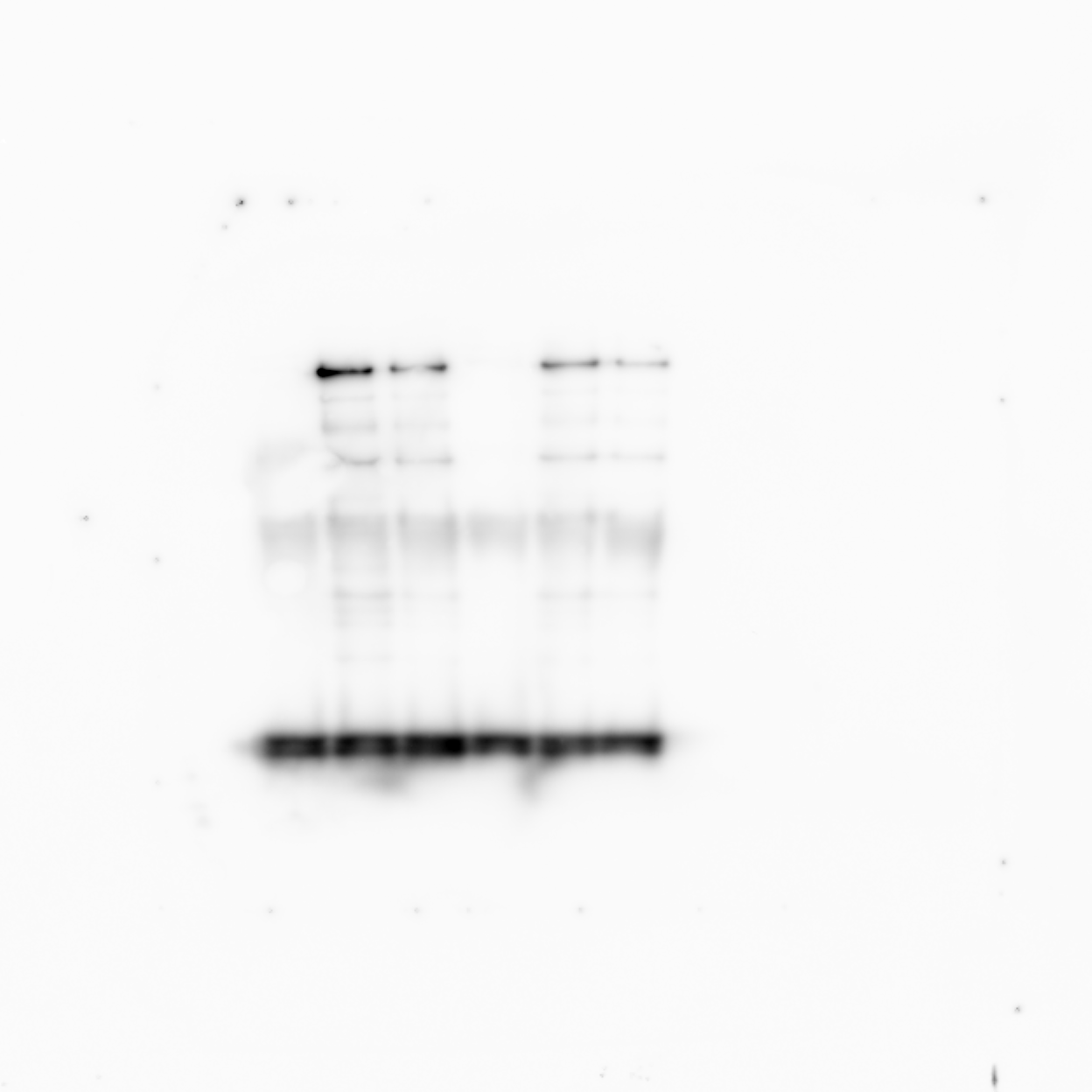

Supplement: Source data 1. [file elife-71662-supp1.zip › Figure 7ΓÇôfigure supplement 2ΓÇôsource data 3.tif]

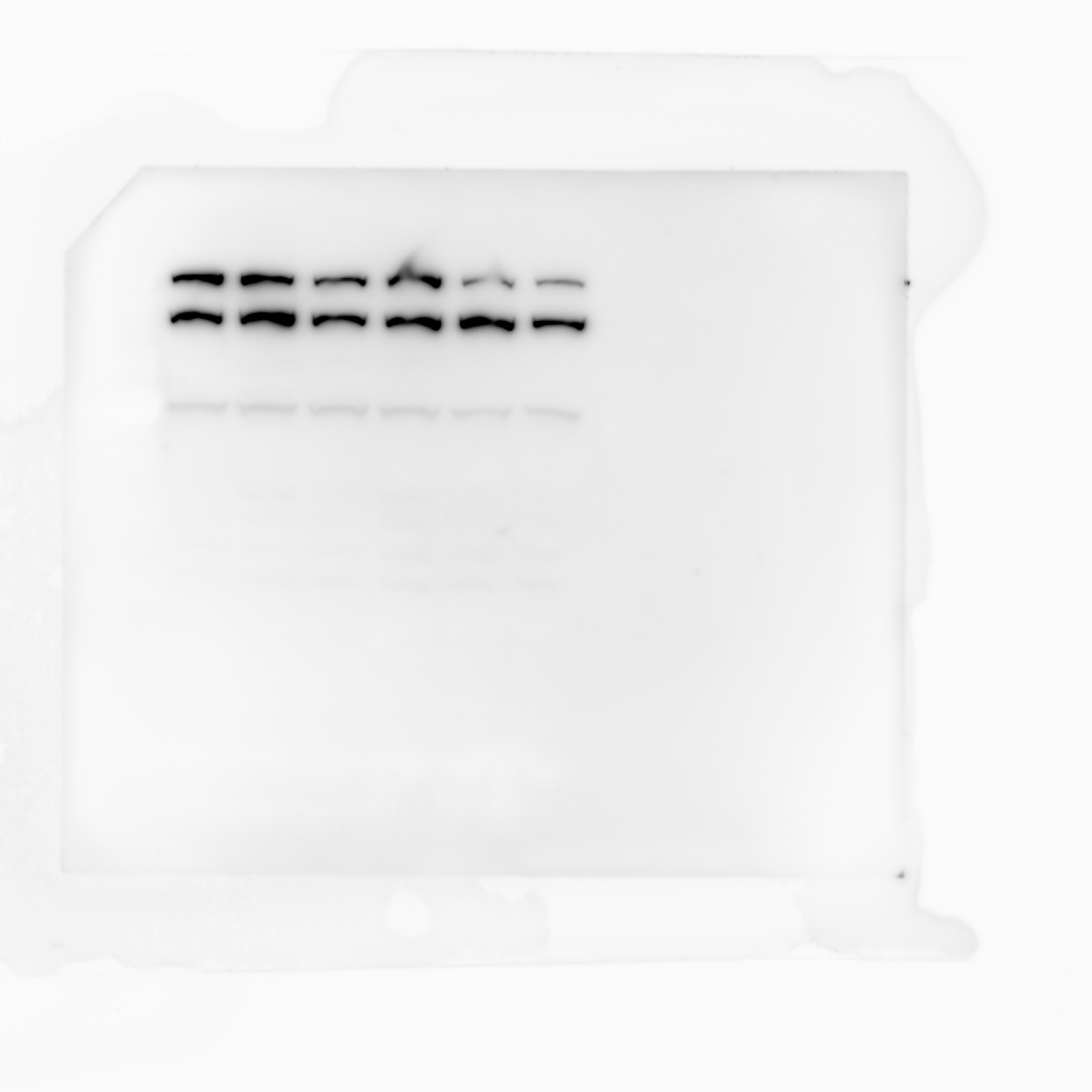

Supplement: Source data 1. [file elife-71662-supp1.zip › Figure 7ΓÇôfigure supplement 2ΓÇôsource data 4.tif]

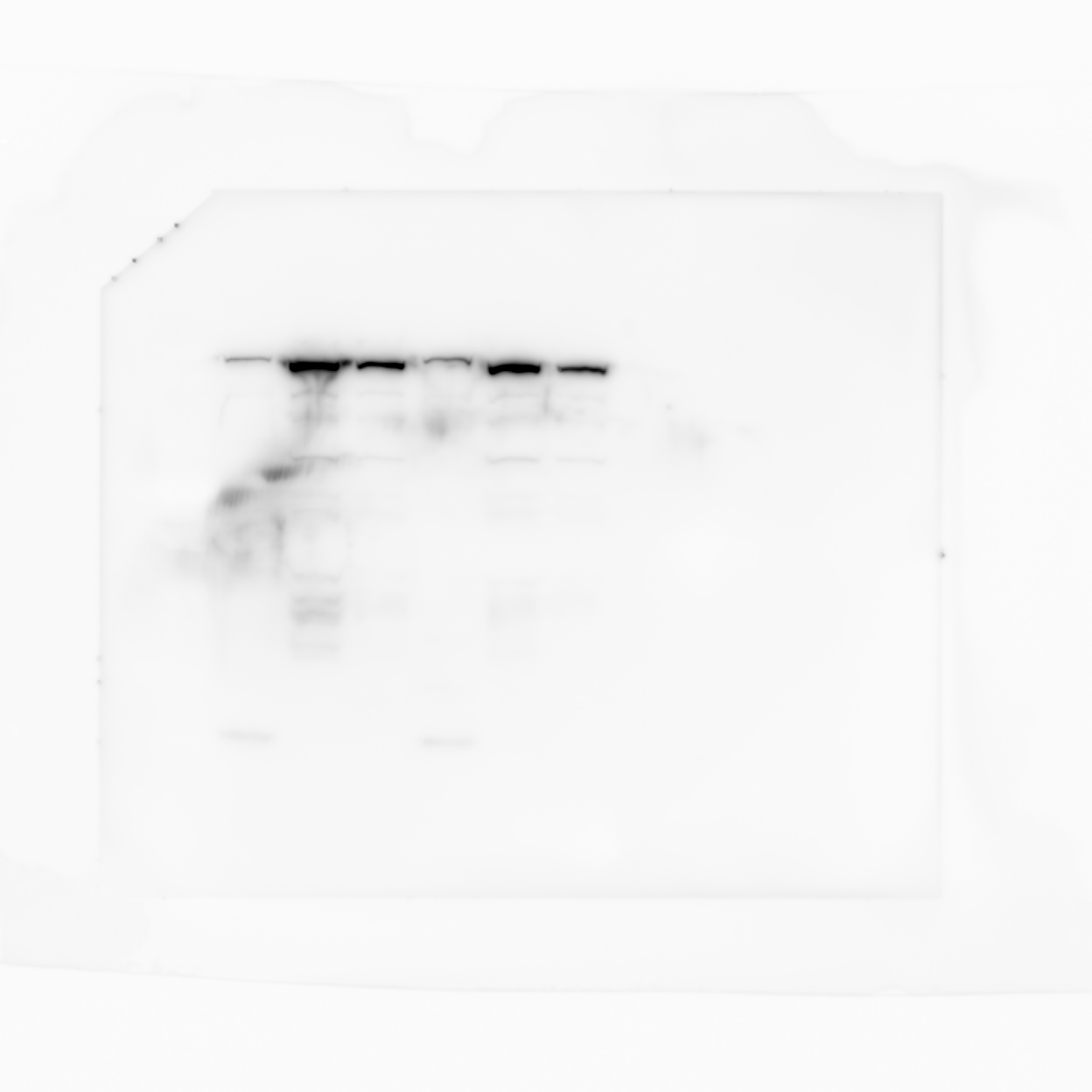

Supplement: Source data 1. [file elife-71662-supp1.zip › Figure 7ΓÇôfigure supplement 2ΓÇôsource data 5.tif]

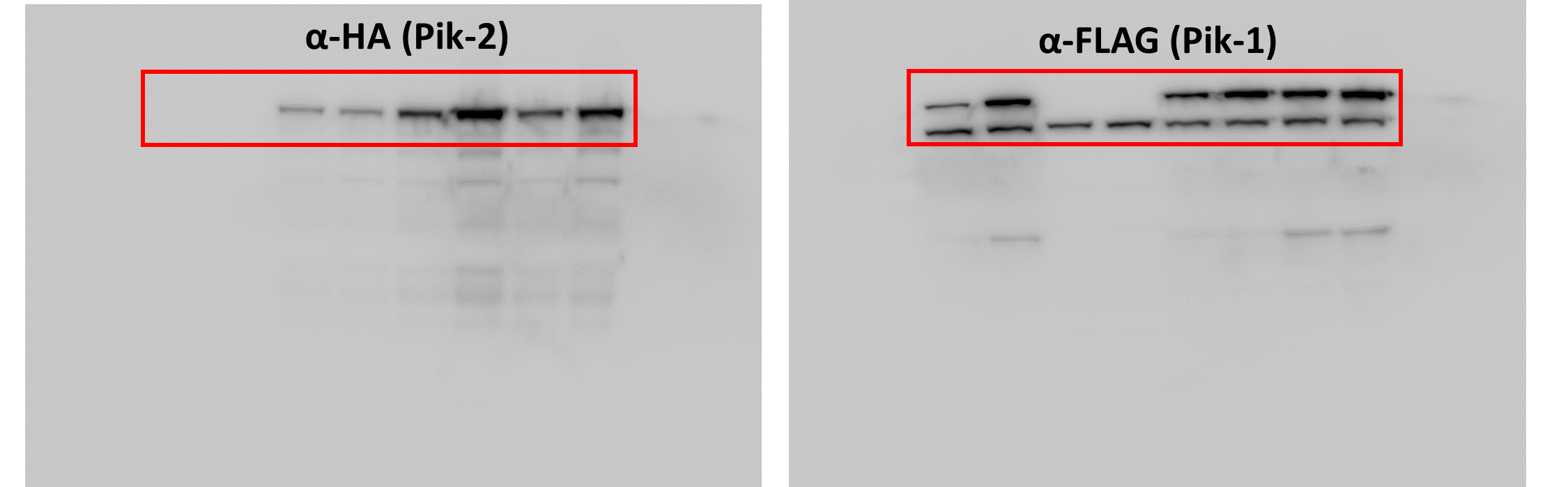

Supplement: Source data 1. [file elife-71662-supp1.zip › Figure 7ΓÇôsource data 1.tif]

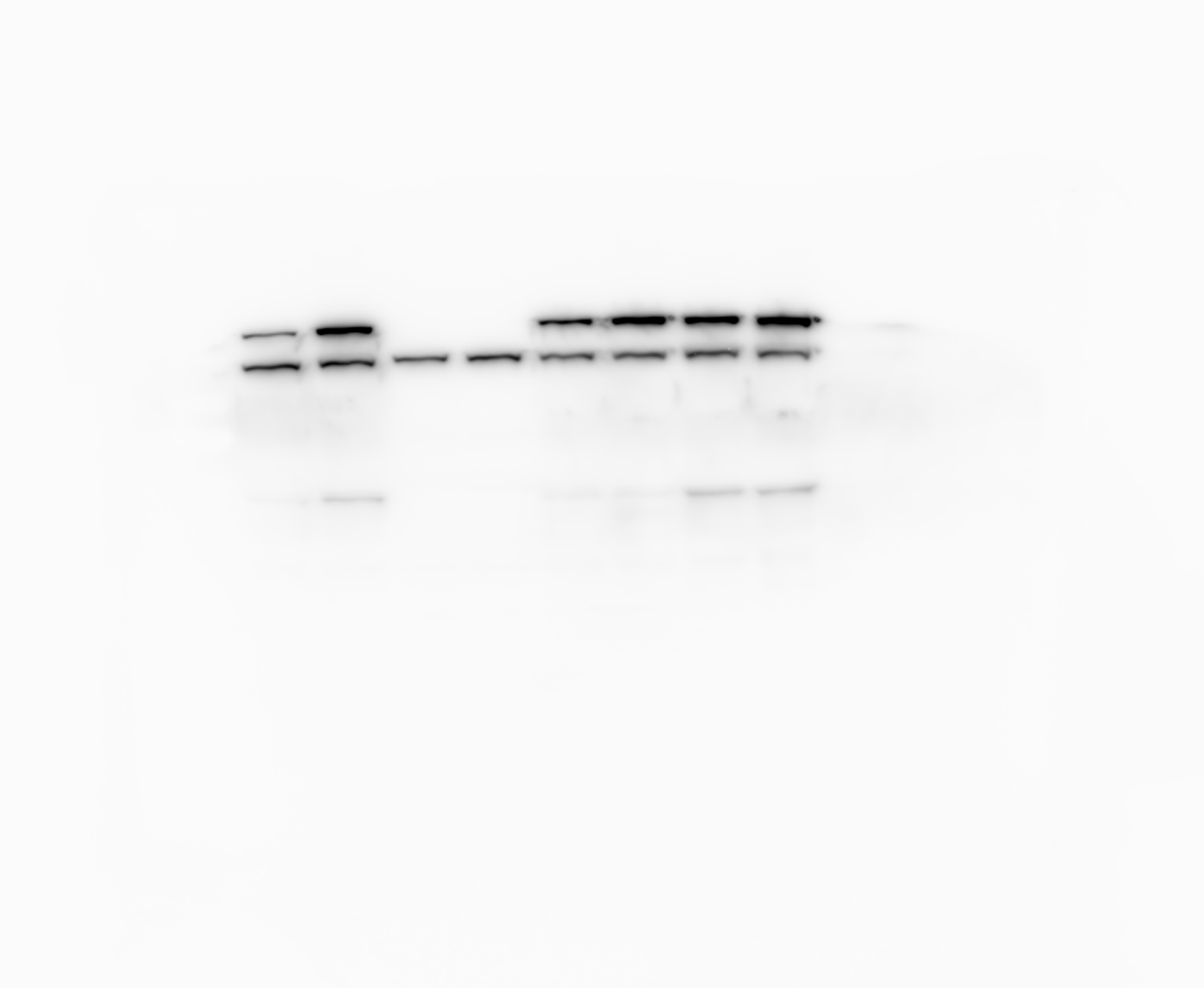

Supplement: Source data 1. [file elife-71662-supp1.zip › Figure 7ΓÇôsource data 2.tif]

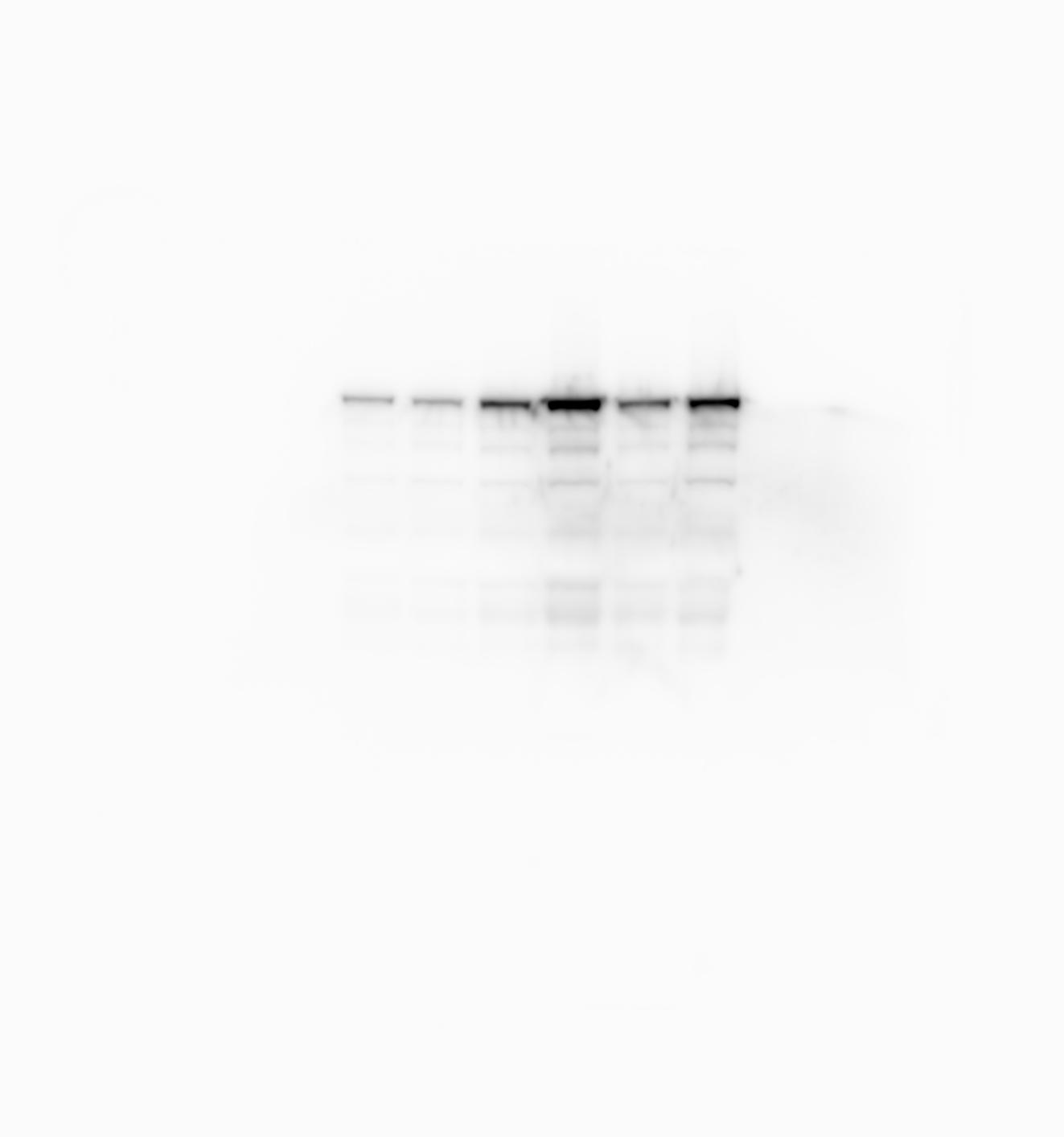

Supplement: Source data 1. [file elife-71662-supp1.zip › Figure 7ΓÇôsource data 3.tif]

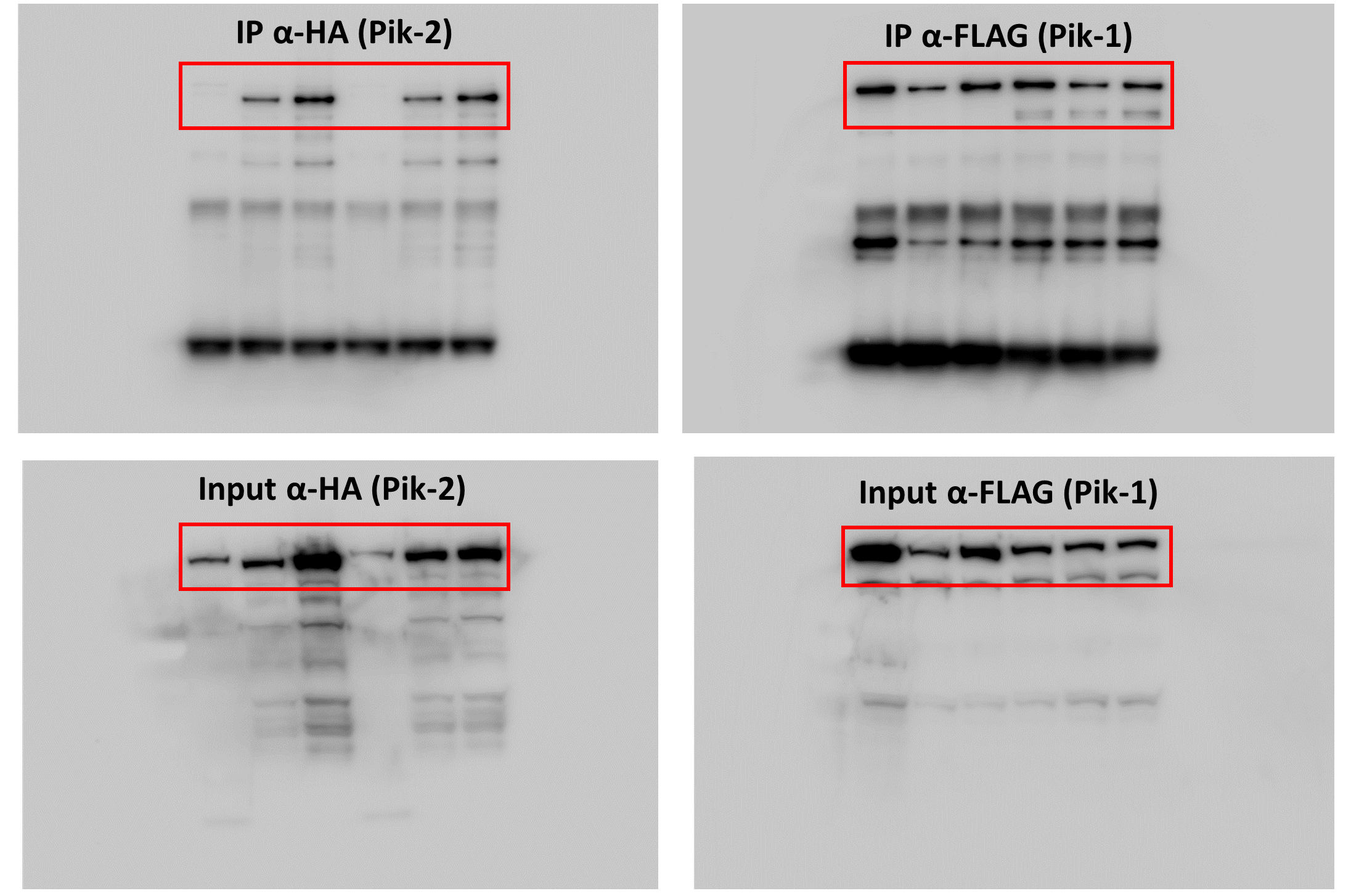

Supplement: Source data 1. [file elife-71662-supp1.zip › Figure 7ΓÇôsource data 4.tif]

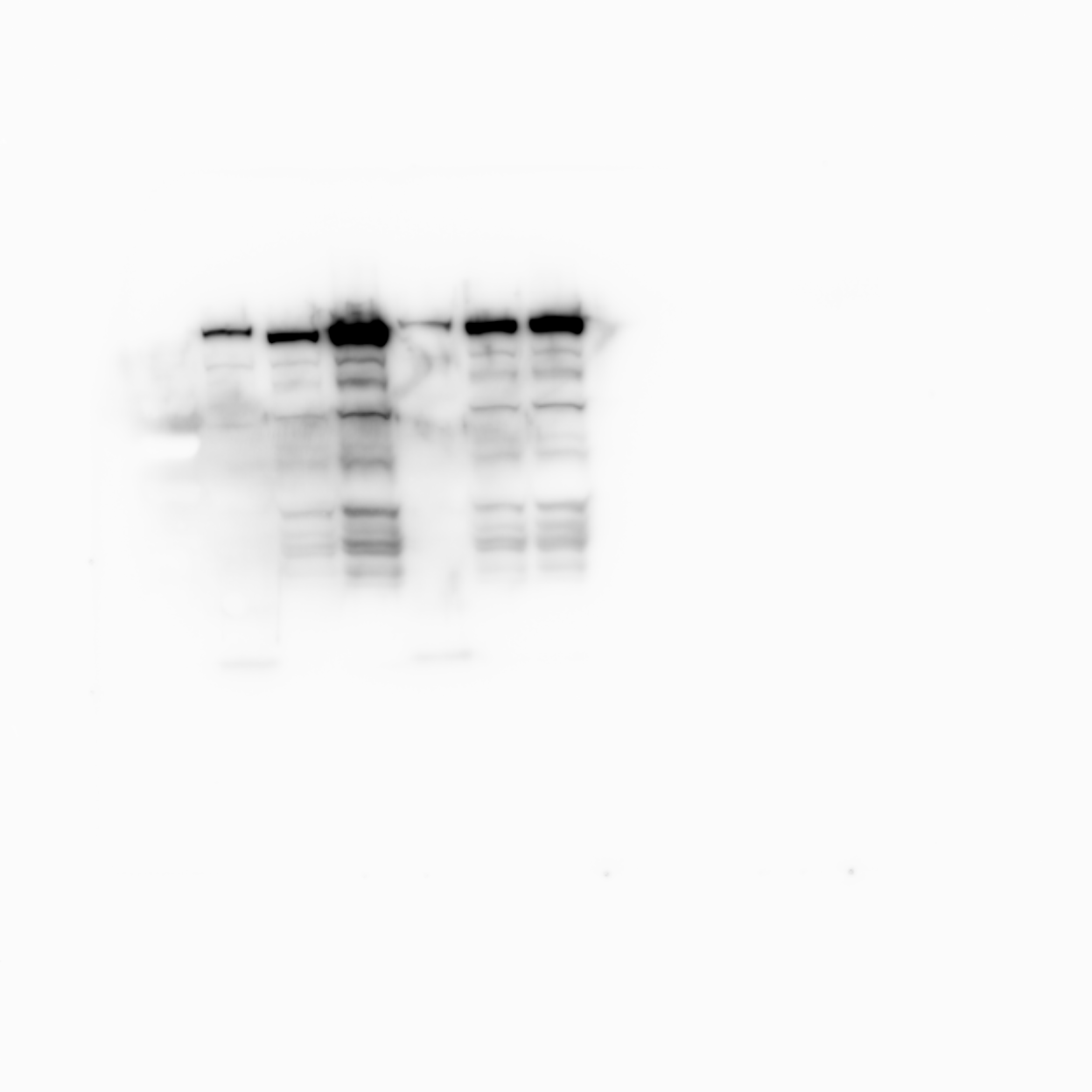

Supplement: Source data 1. [file elife-71662-supp1.zip › Figure 7ΓÇôsource data 5.tif]

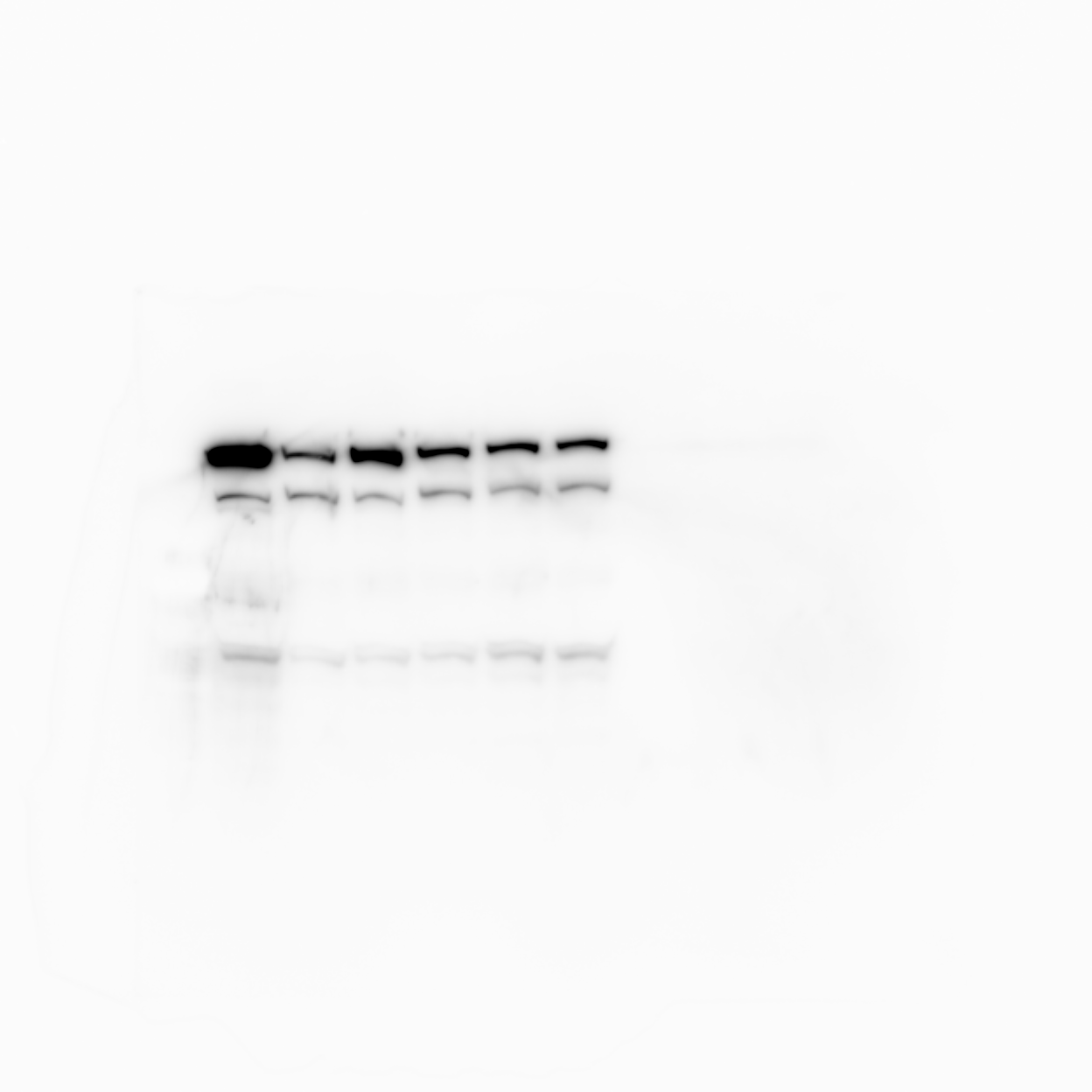

Supplement: Source data 1. [file elife-71662-supp1.zip › Figure 7ΓÇôsource data 6.tif]

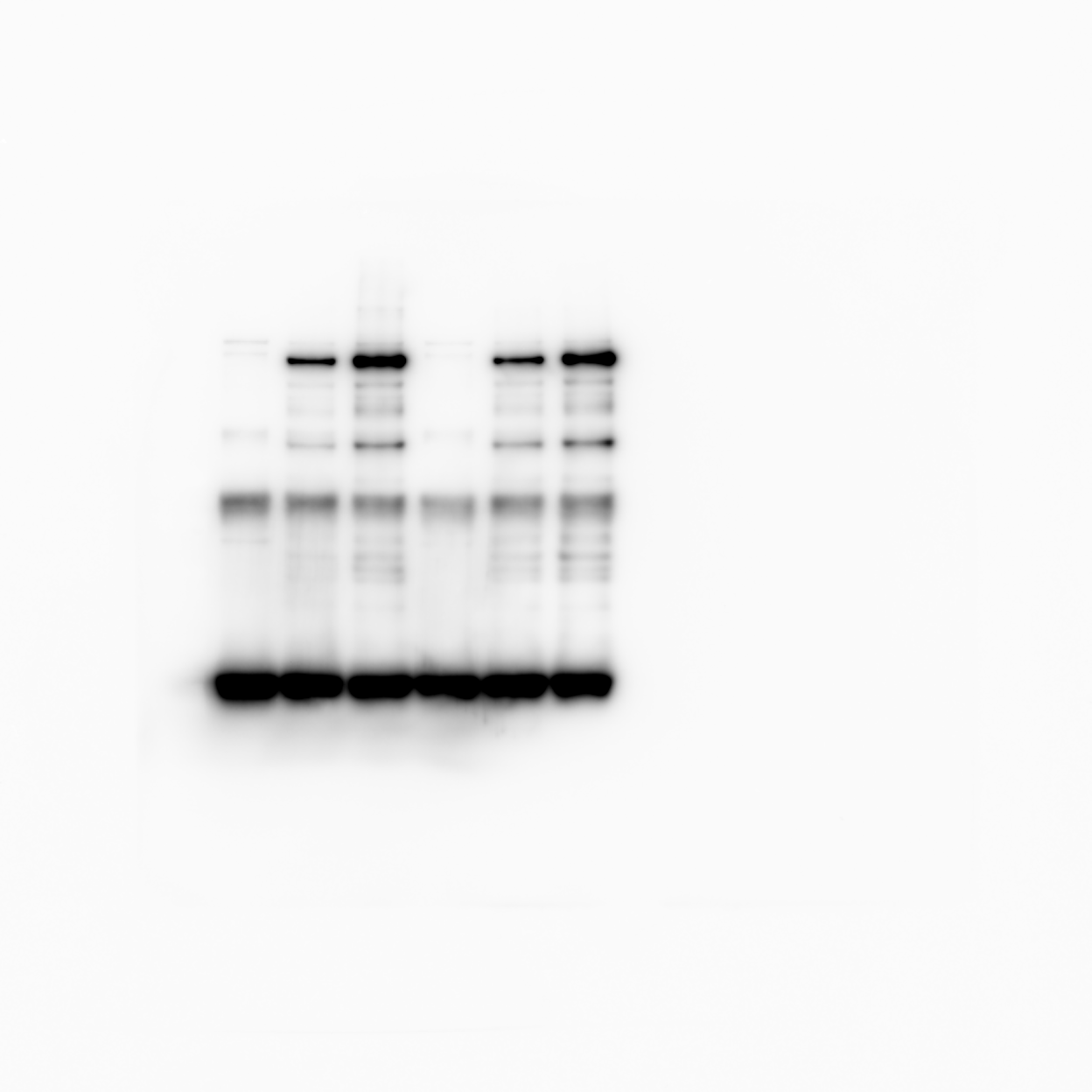

Supplement: Source data 1. [file elife-71662-supp1.zip › Figure 7ΓÇôsource data 7.tif]

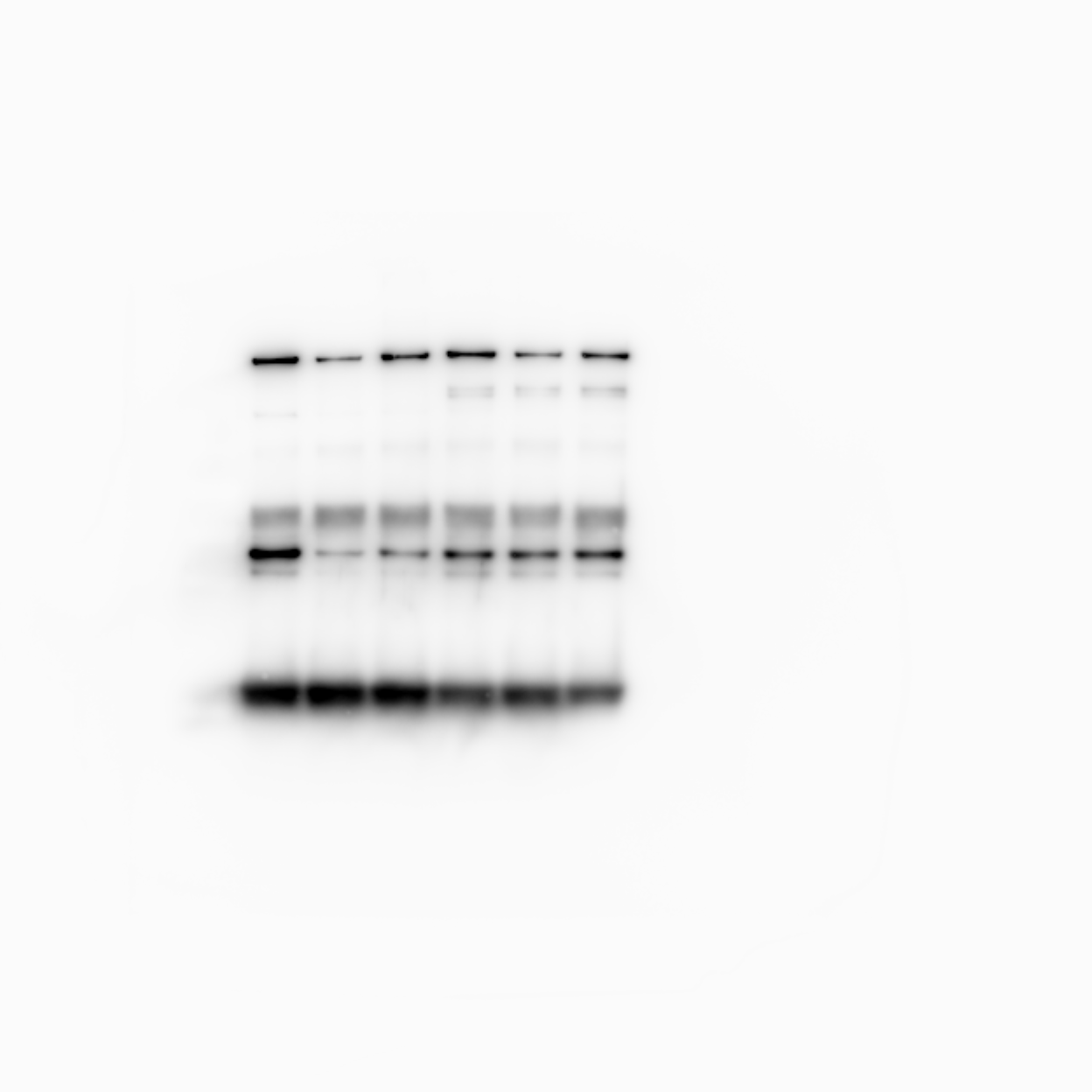

Supplement: Source data 1. [file elife-71662-supp1.zip › Figure 7ΓÇôsource data 8.tif]

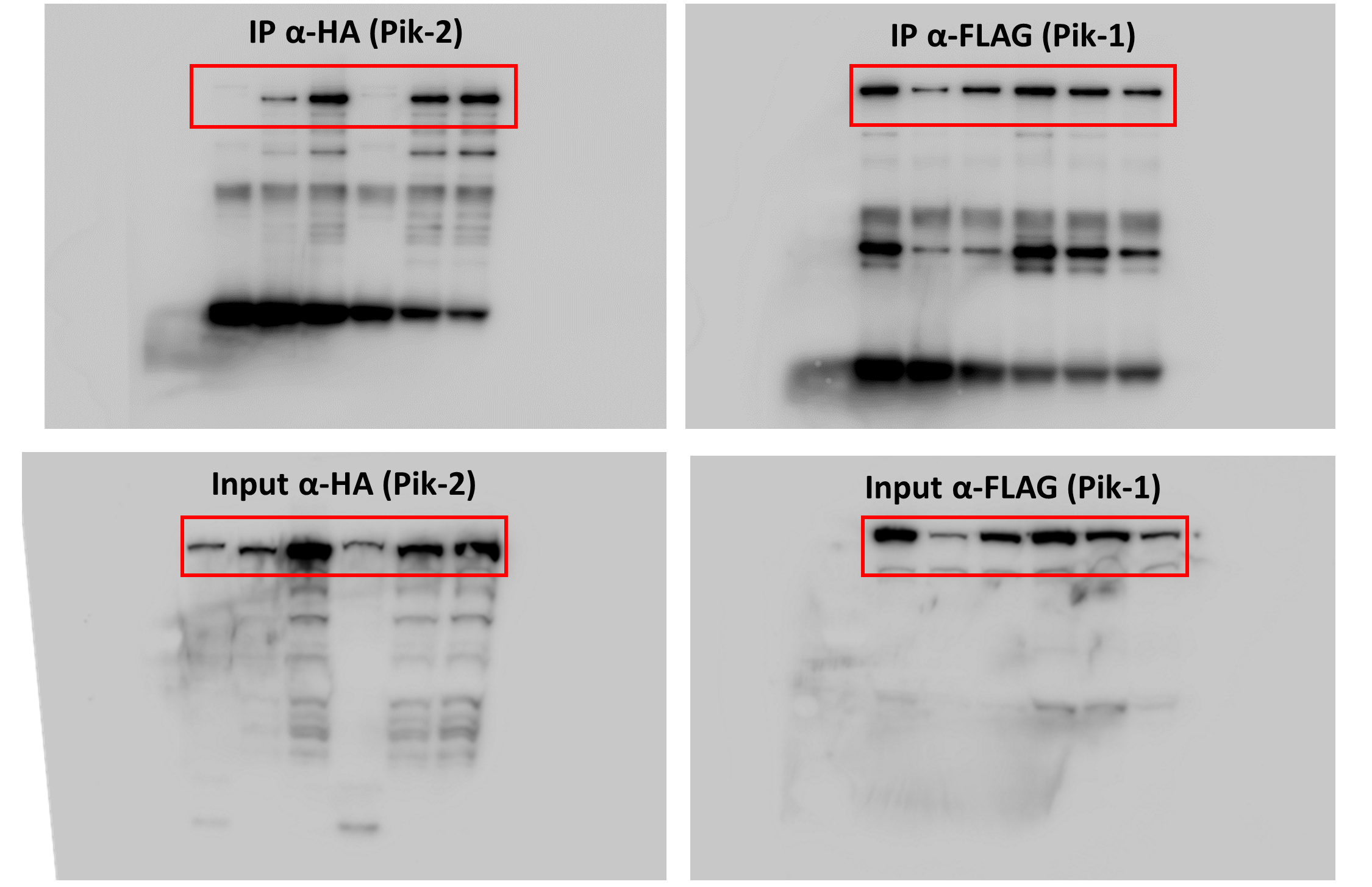

Supplement: Source data 1. [file elife-71662-supp1.zip › Figure 8ΓÇôfigure supplement 1ΓÇôsource data 1.tif]

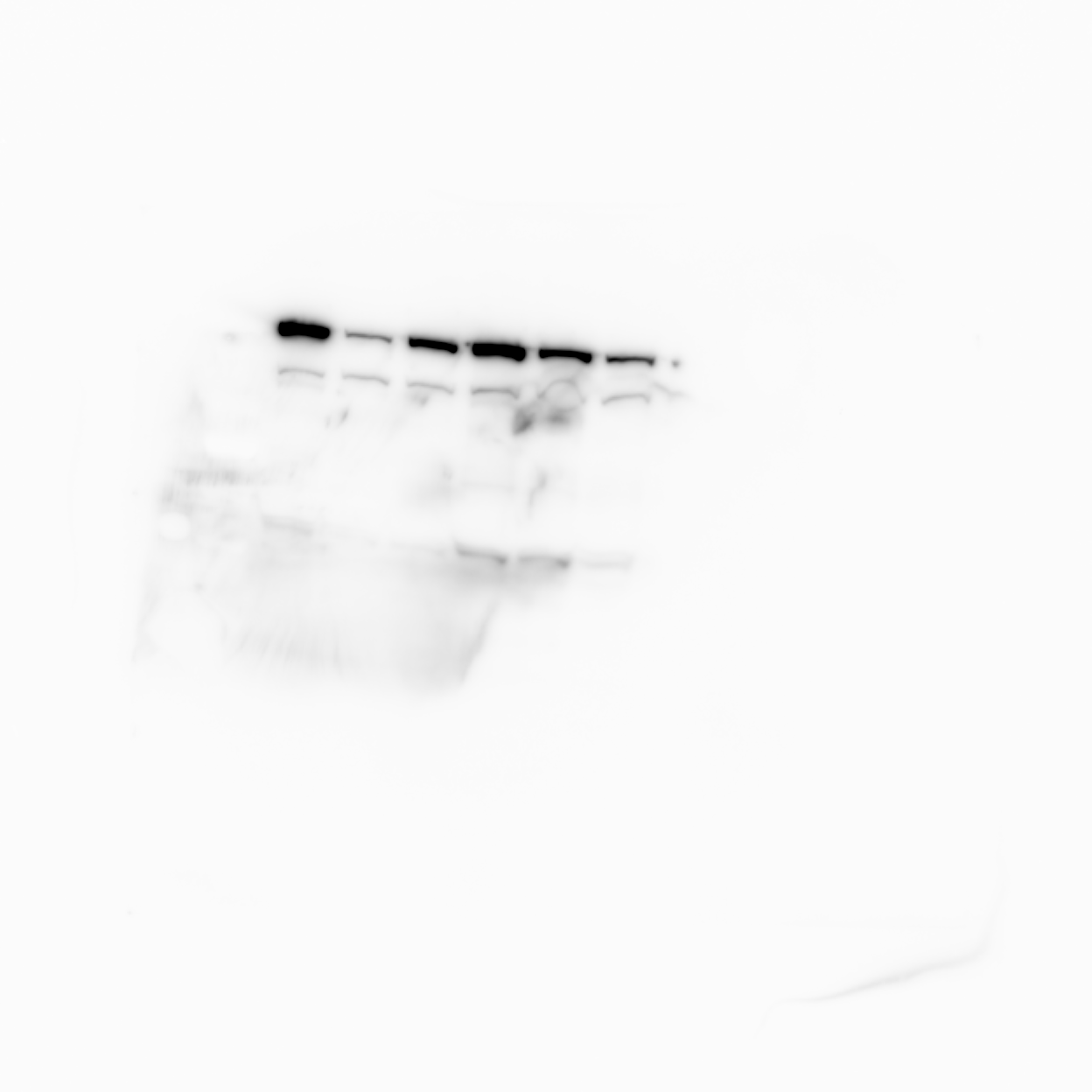

Supplement: Source data 1. [file elife-71662-supp1.zip › Figure 8ΓÇôfigure supplement 1ΓÇôsource data 2.tif]

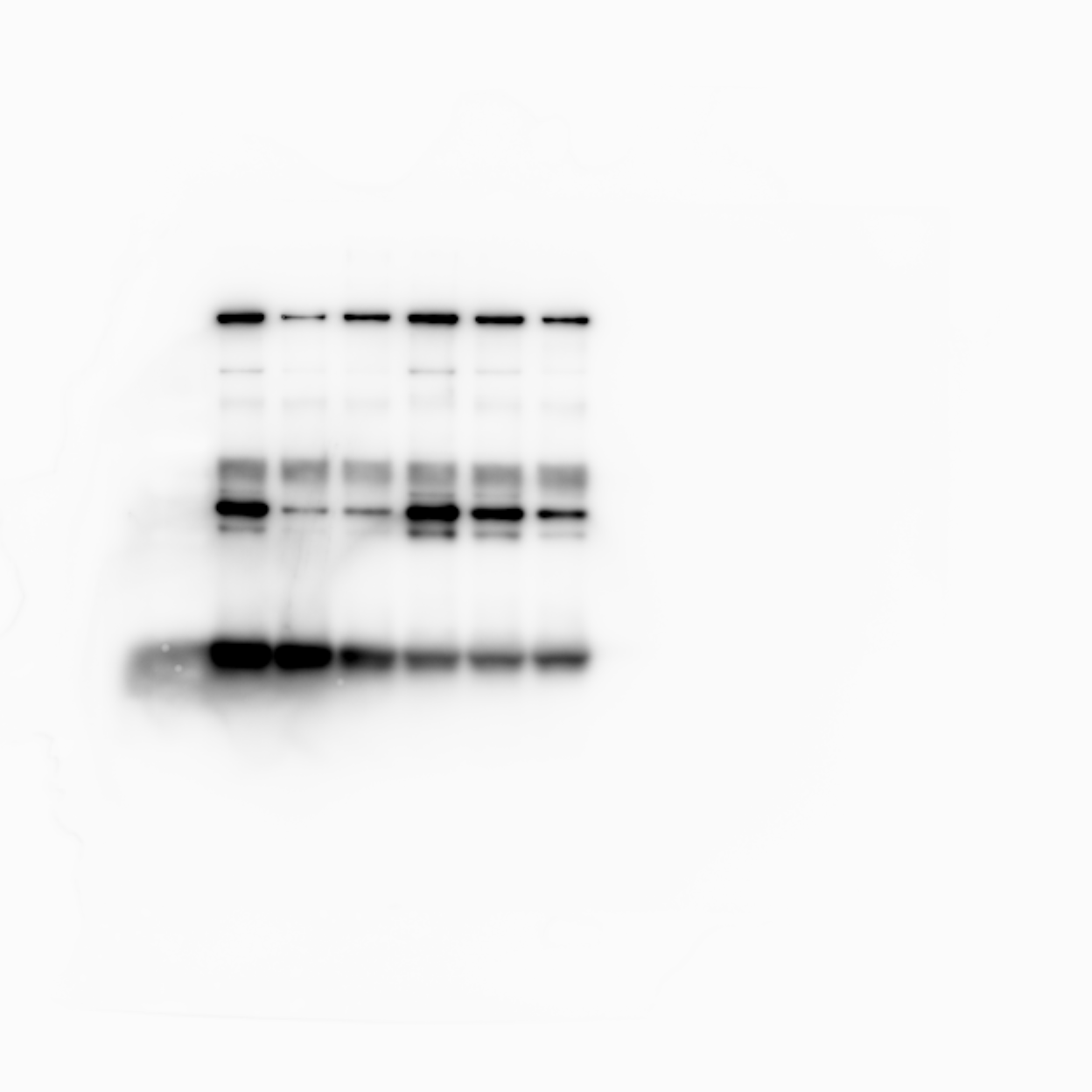

Supplement: Source data 1. [file elife-71662-supp1.zip › Figure 8ΓÇôfigure supplement 1ΓÇôsource data 3.tif]

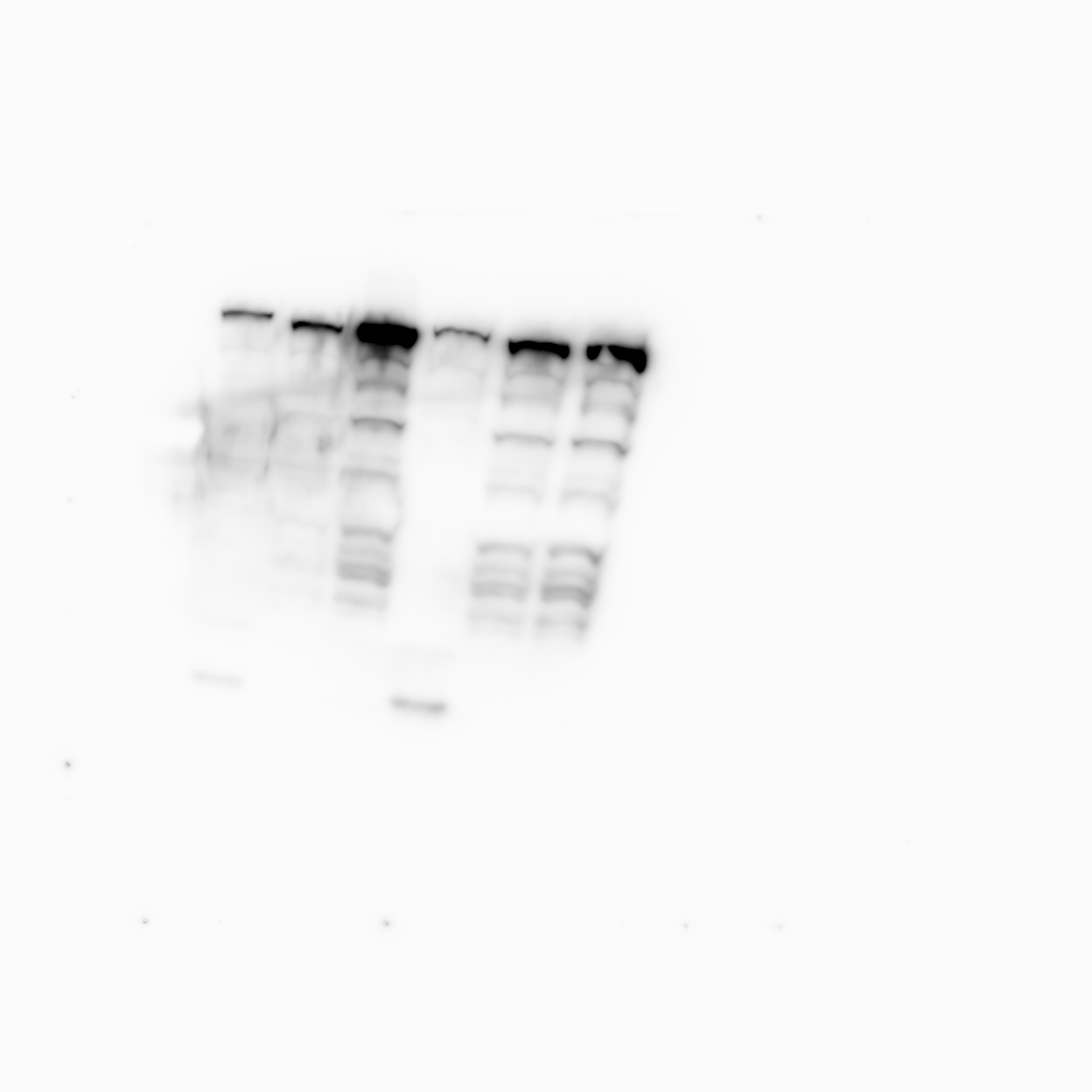

Supplement: Source data 1. [file elife-71662-supp1.zip › Figure 8ΓÇôfigure supplement 1ΓÇôsource data 4.tif]

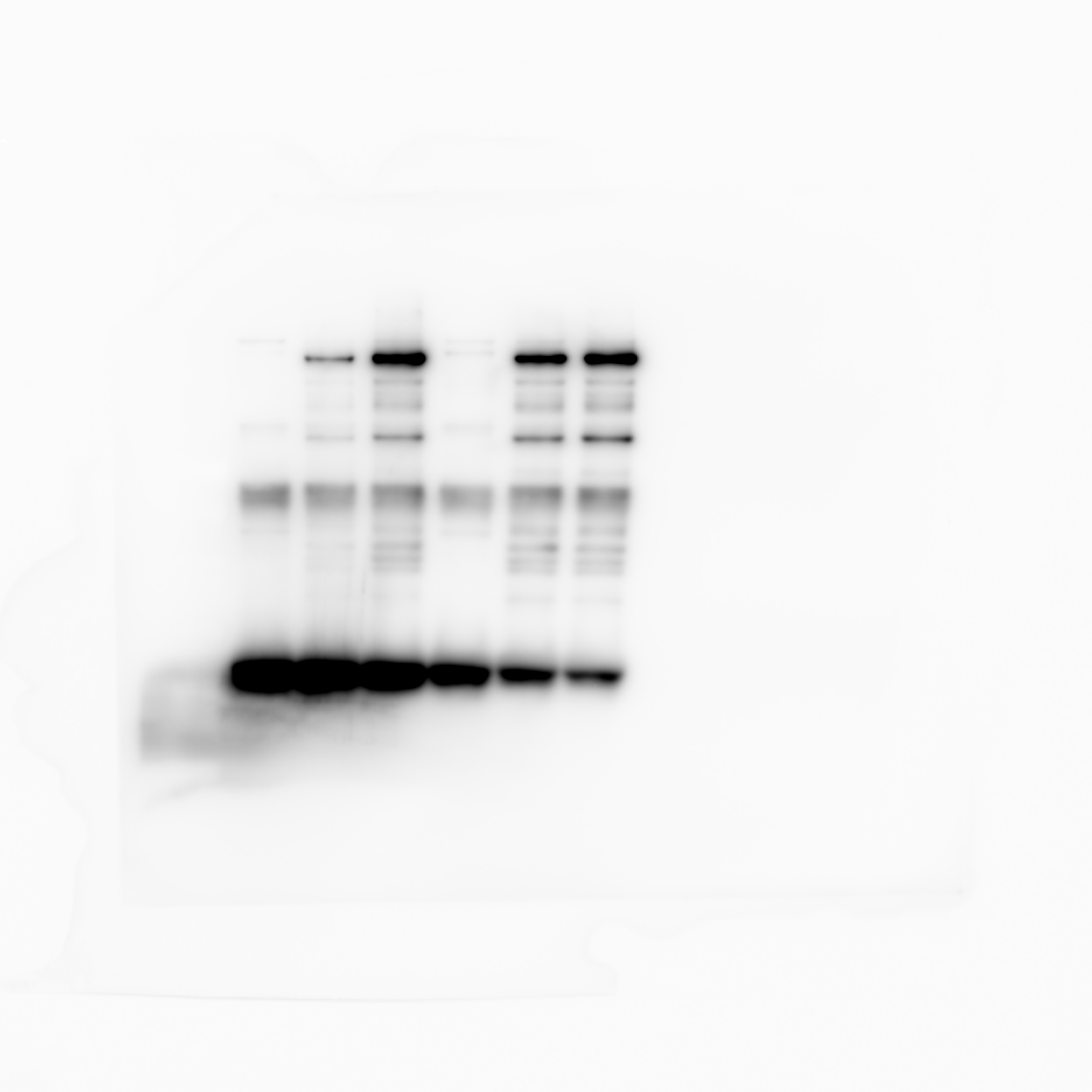

Supplement: Source data 1. [file elife-71662-supp1.zip › Figure 8ΓÇôfigure supplement 1ΓÇôsource data 5.tif]

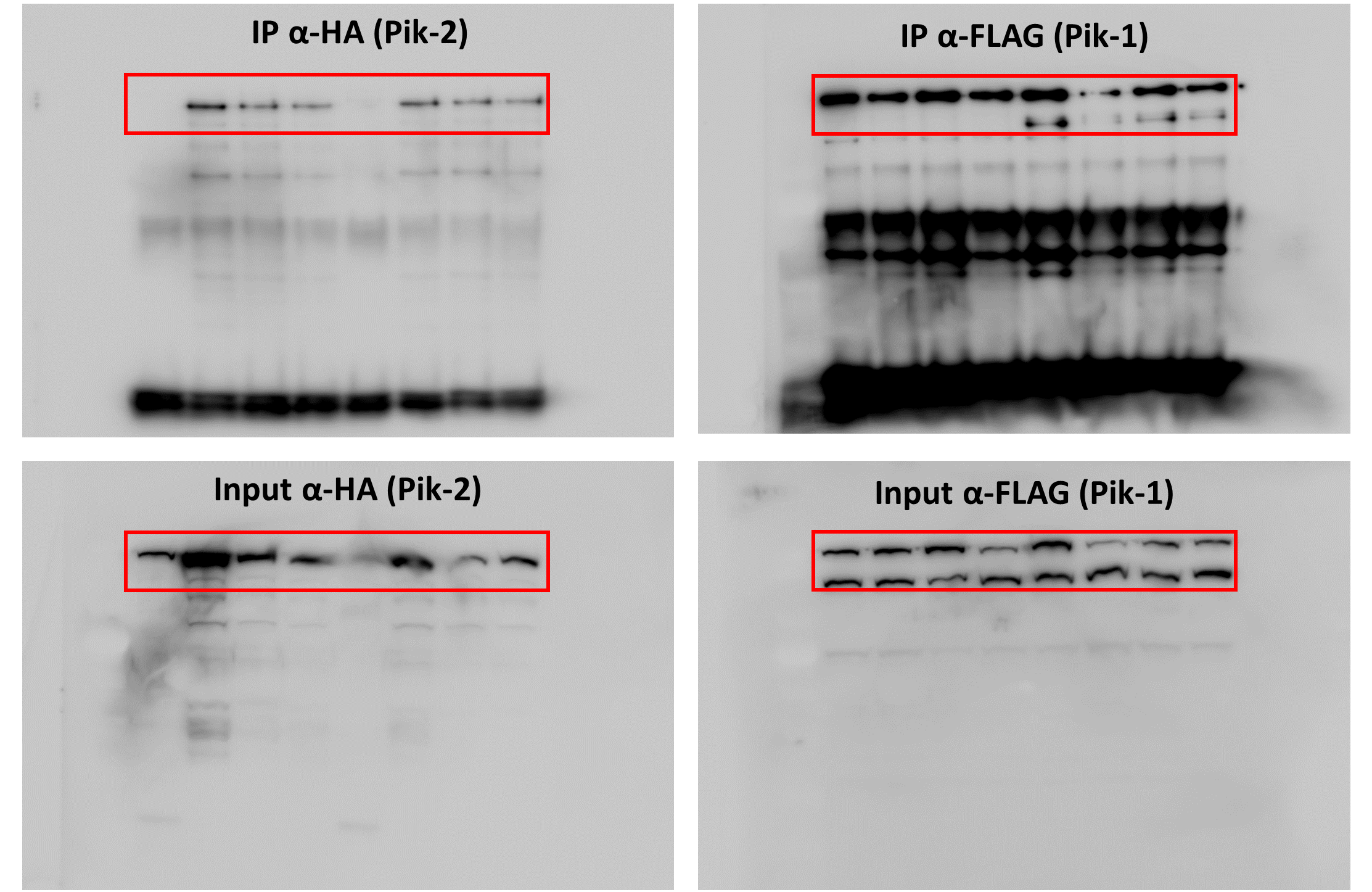

Supplement: Source data 1. [file elife-71662-supp1.zip › Figure 8ΓÇôsource data 1.tif]

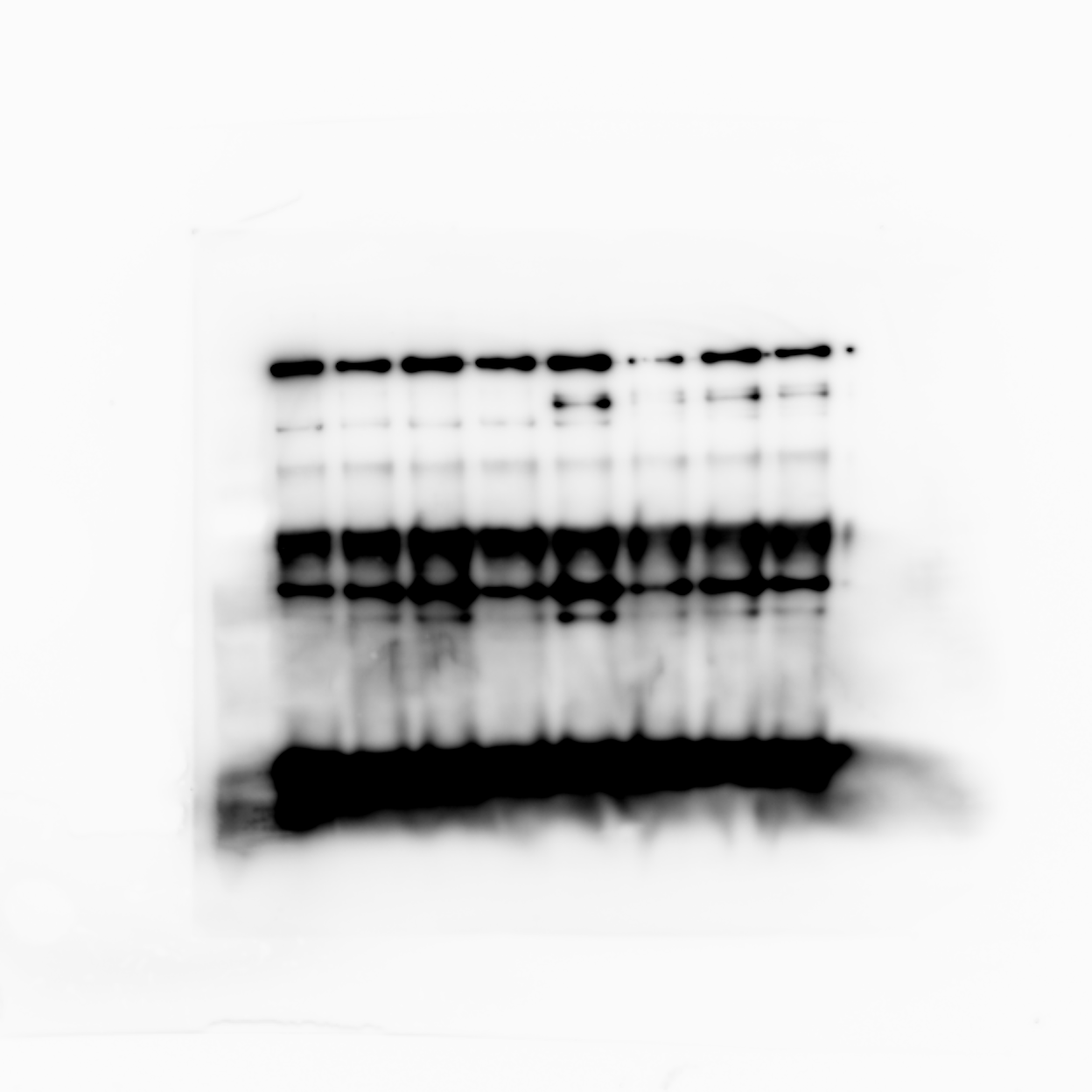

Supplement: Source data 1. [file elife-71662-supp1.zip › Figure 8ΓÇôsource data 2.tif]

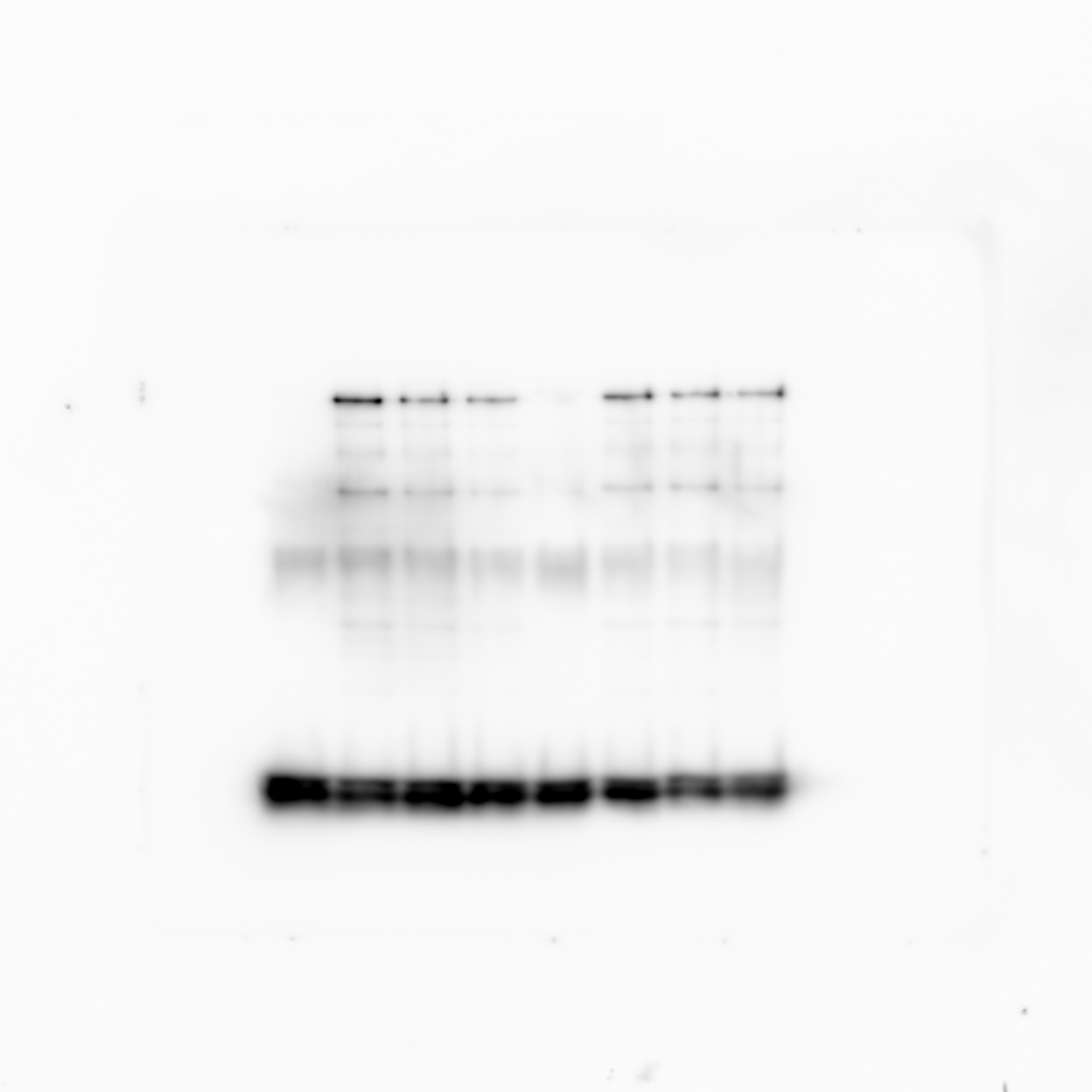

Supplement: Source data 1. [file elife-71662-supp1.zip › Figure 8ΓÇôsource data 3.tif]

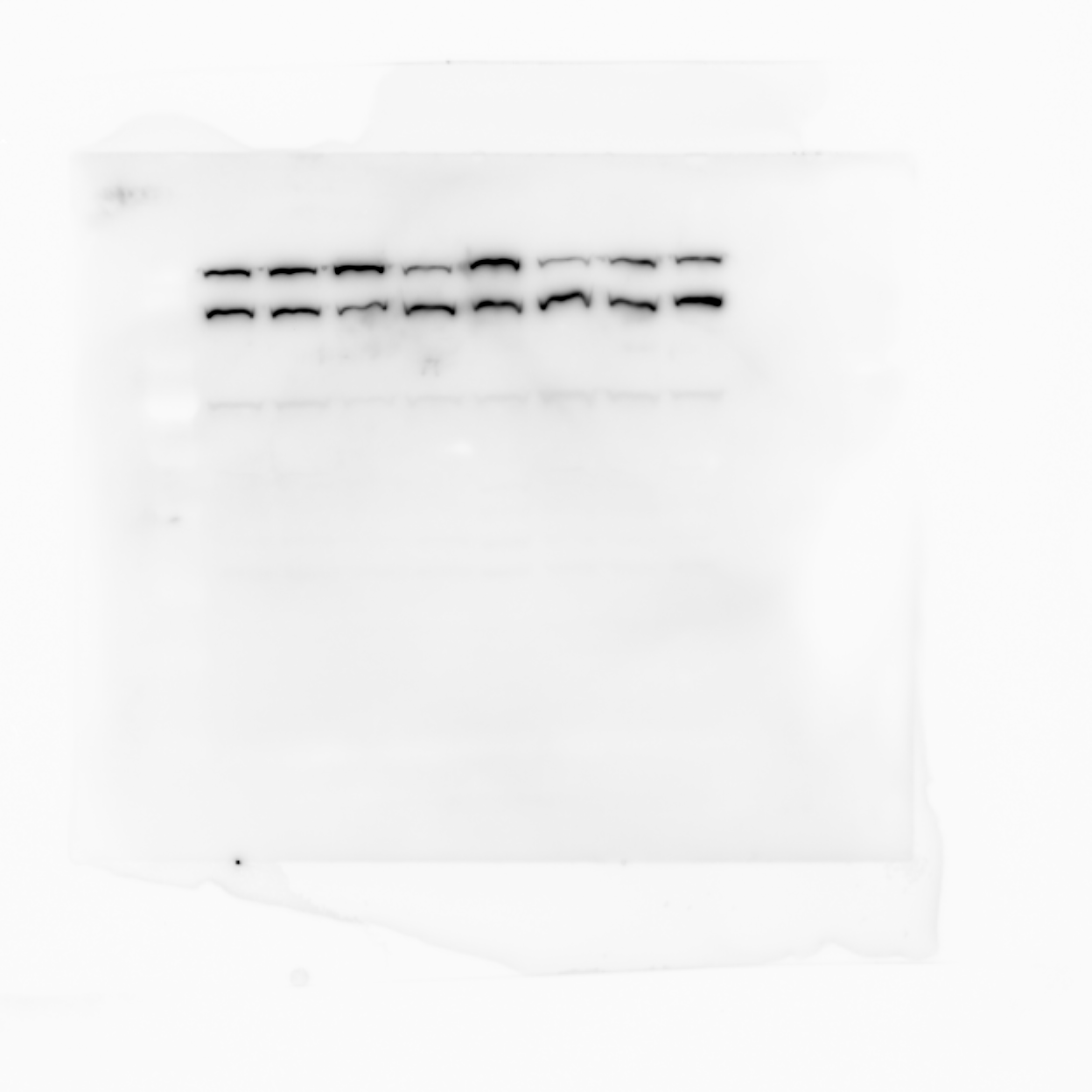

Supplement: Source data 1. [file elife-71662-supp1.zip › Figure 8ΓÇôsource data 4.tif]

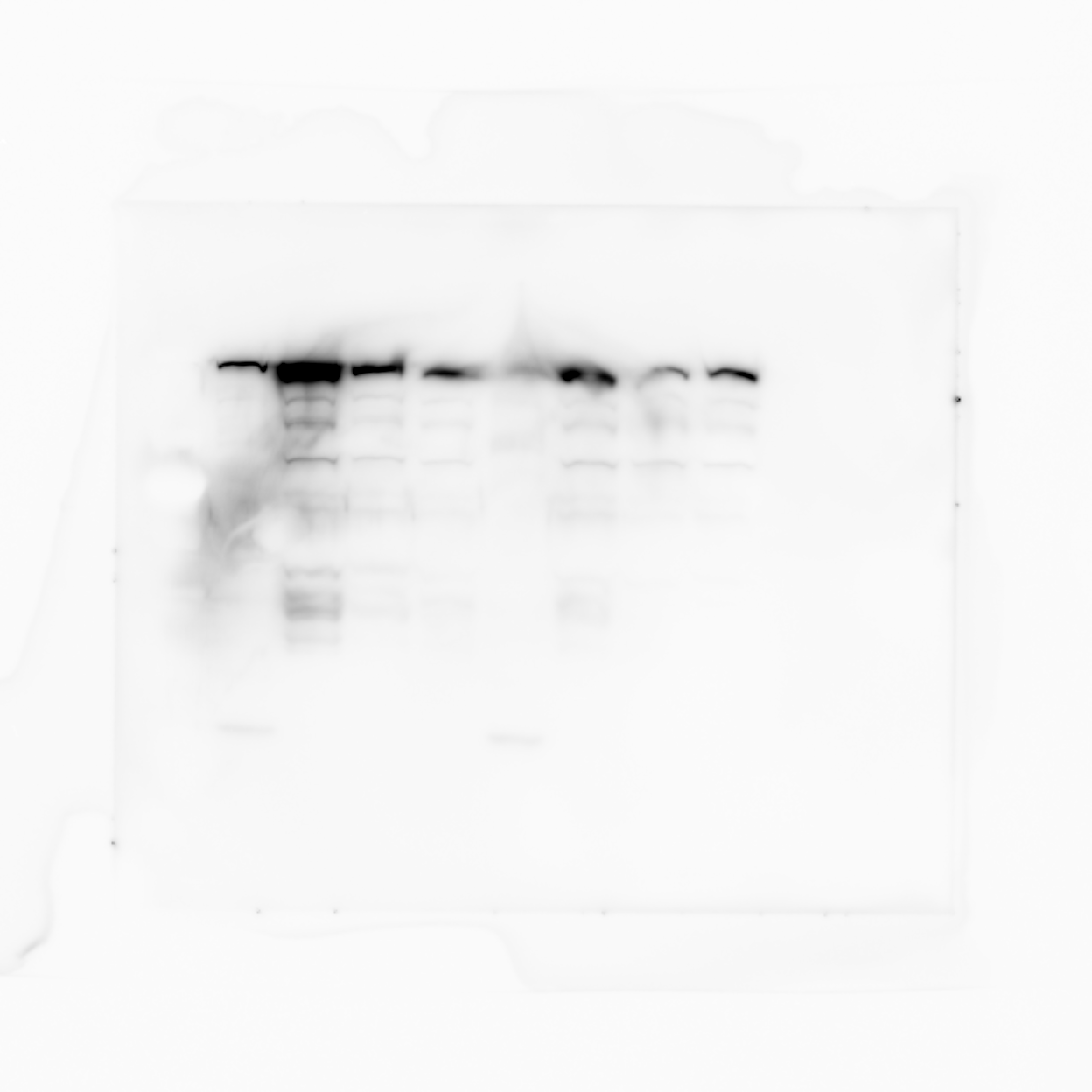

Supplement: Source data 1. [file elife-71662-supp1.zip › Figure 8ΓÇôsource data 5.tif]

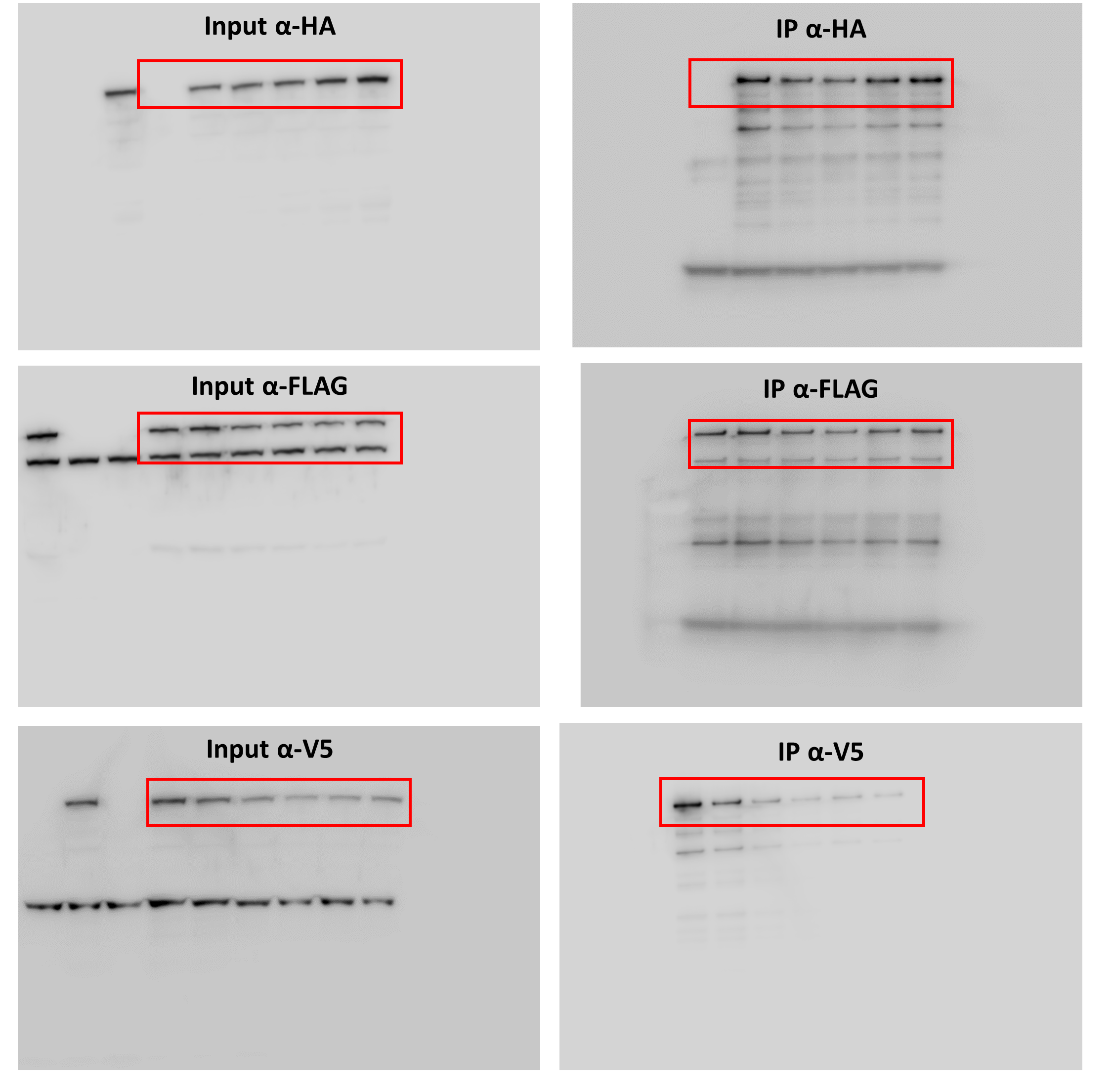

Supplement: Source data 1. [file elife-71662-supp1.zip › Figure 9ΓÇôsource data 1.tif]

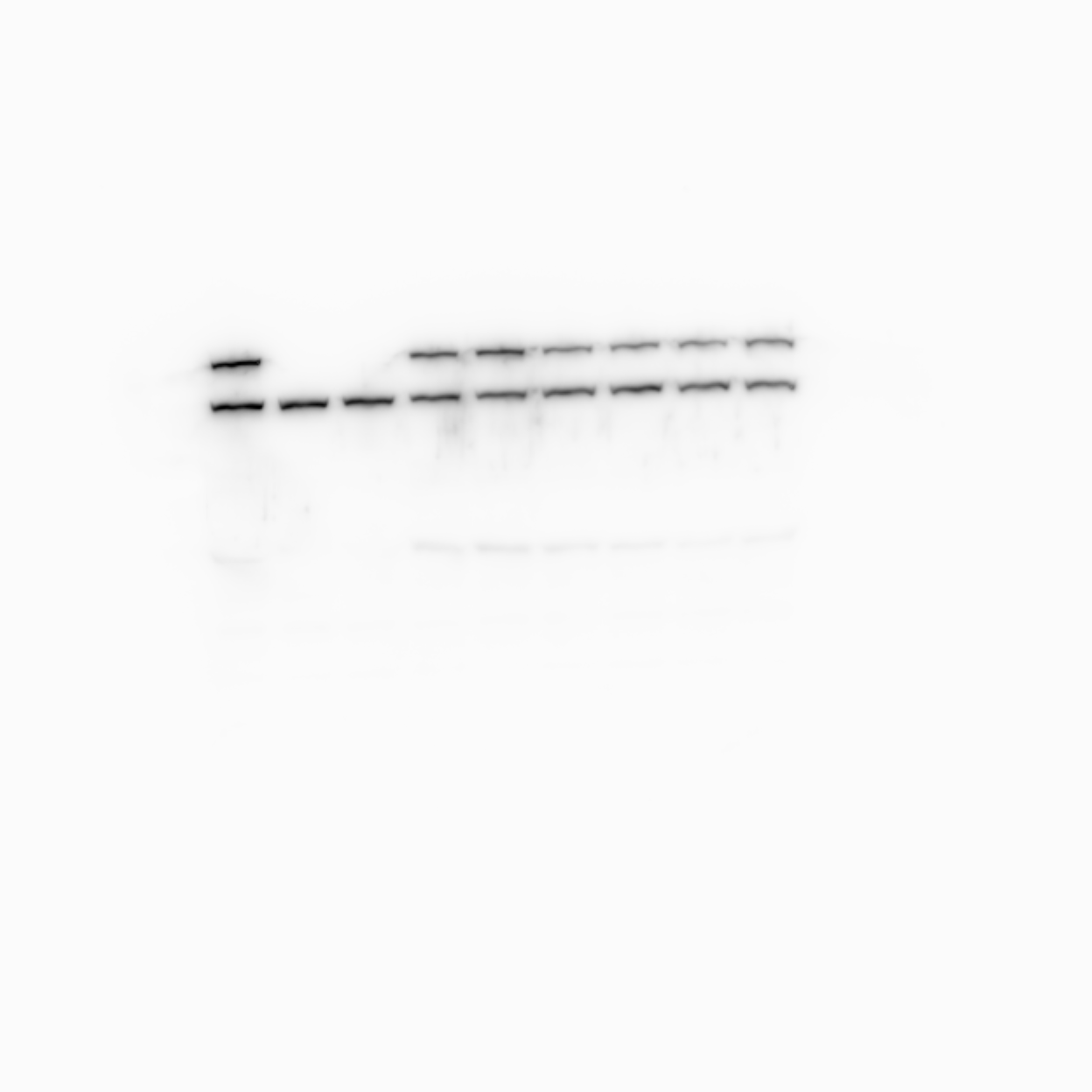

Supplement: Source data 1. [file elife-71662-supp1.zip › Figure 9ΓÇôsource data 2.tif]

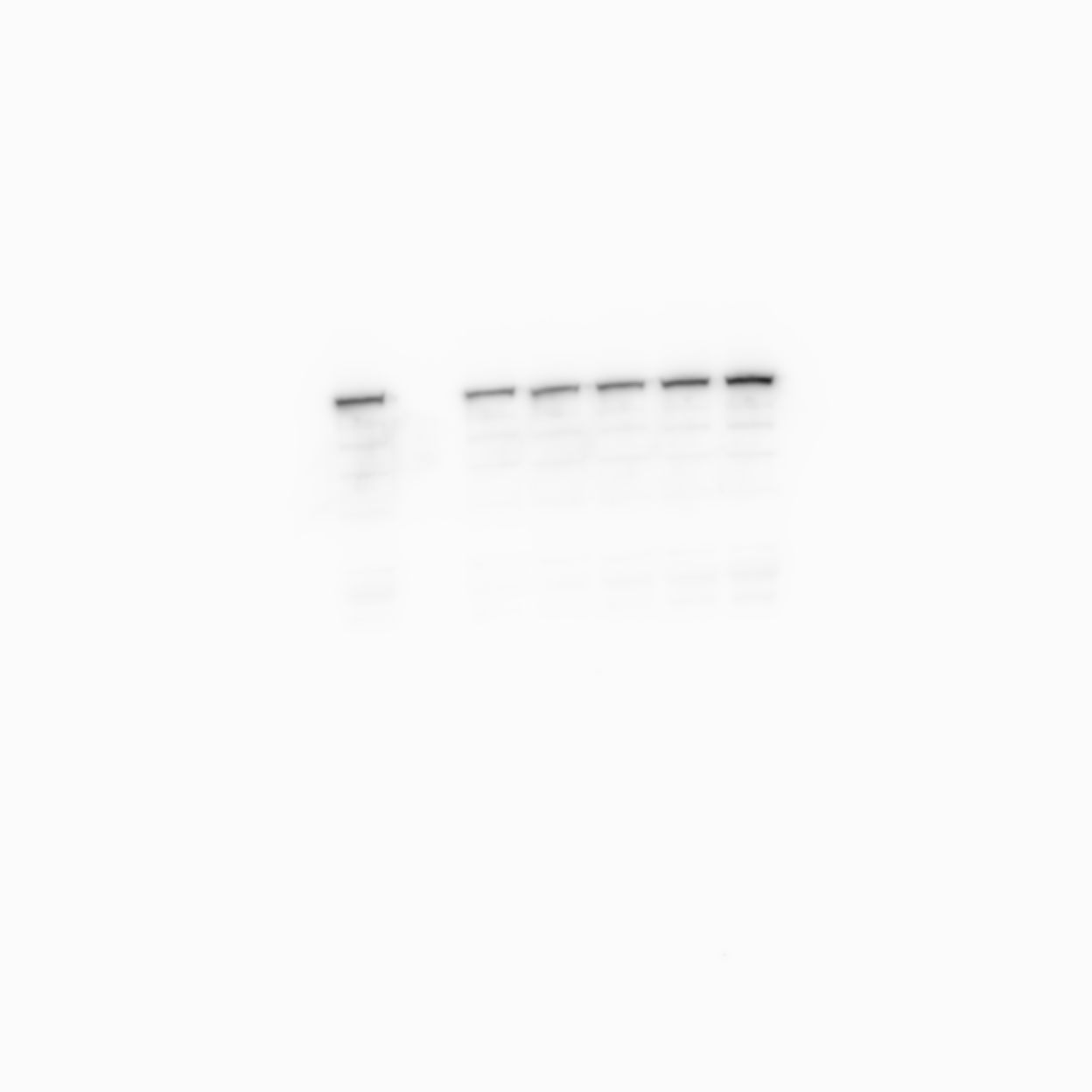

Supplement: Source data 1. [file elife-71662-supp1.zip › Figure 9ΓÇôsource data 3.tif]

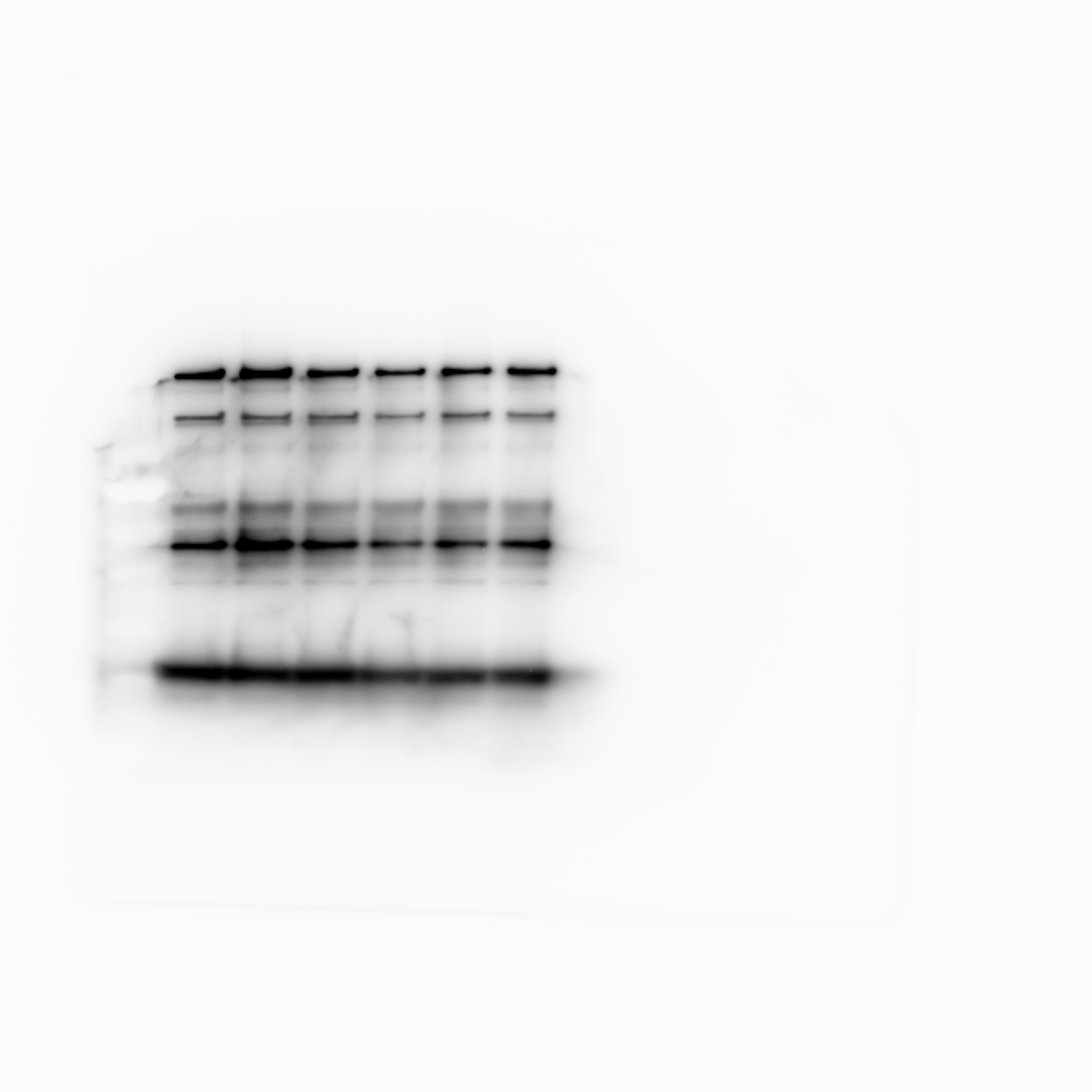

Supplement: Source data 1. [file elife-71662-supp1.zip › Figure 9ΓÇôsource data 4.tif]

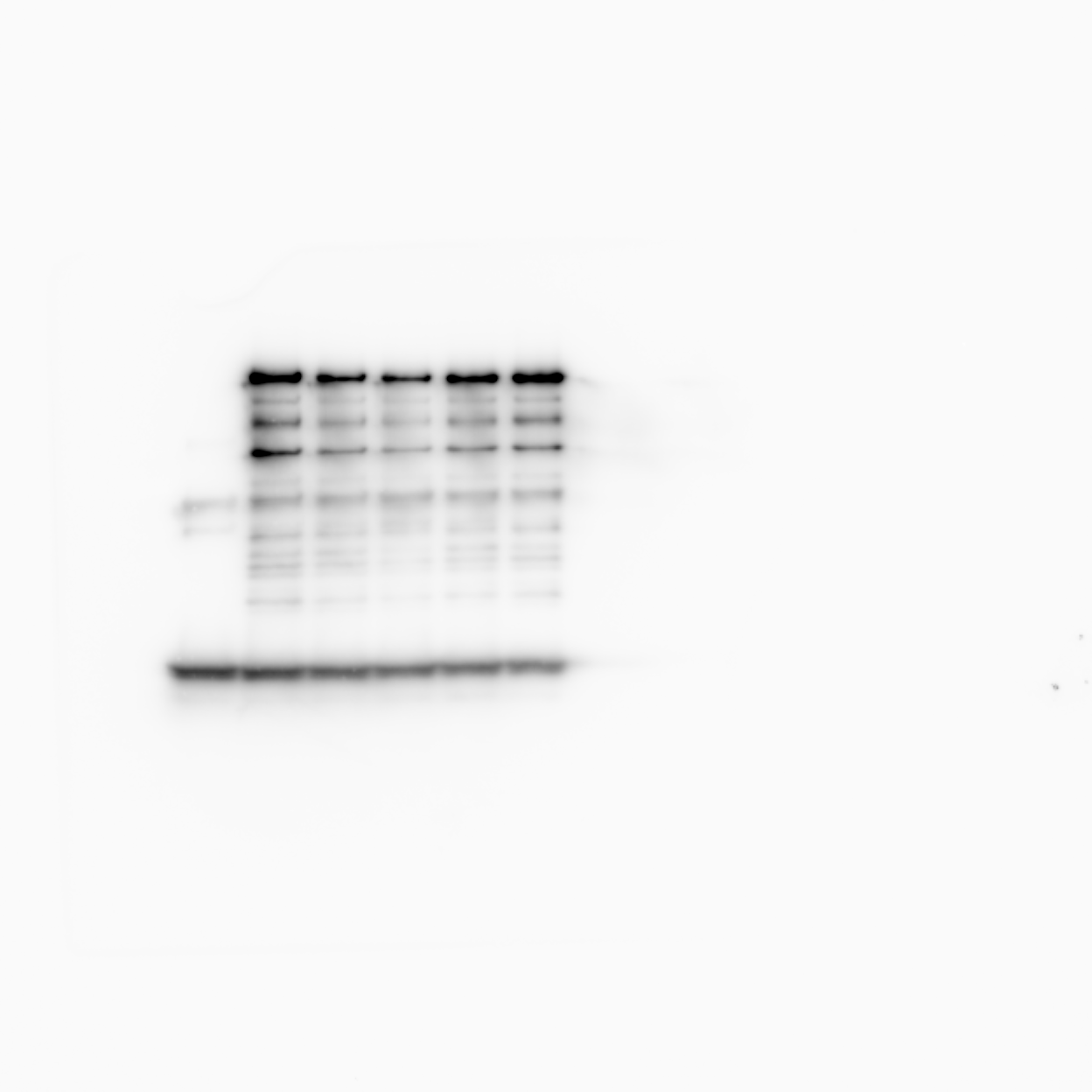

Supplement: Source data 1. [file elife-71662-supp1.zip › Figure 9ΓÇôsource data 5.tif]

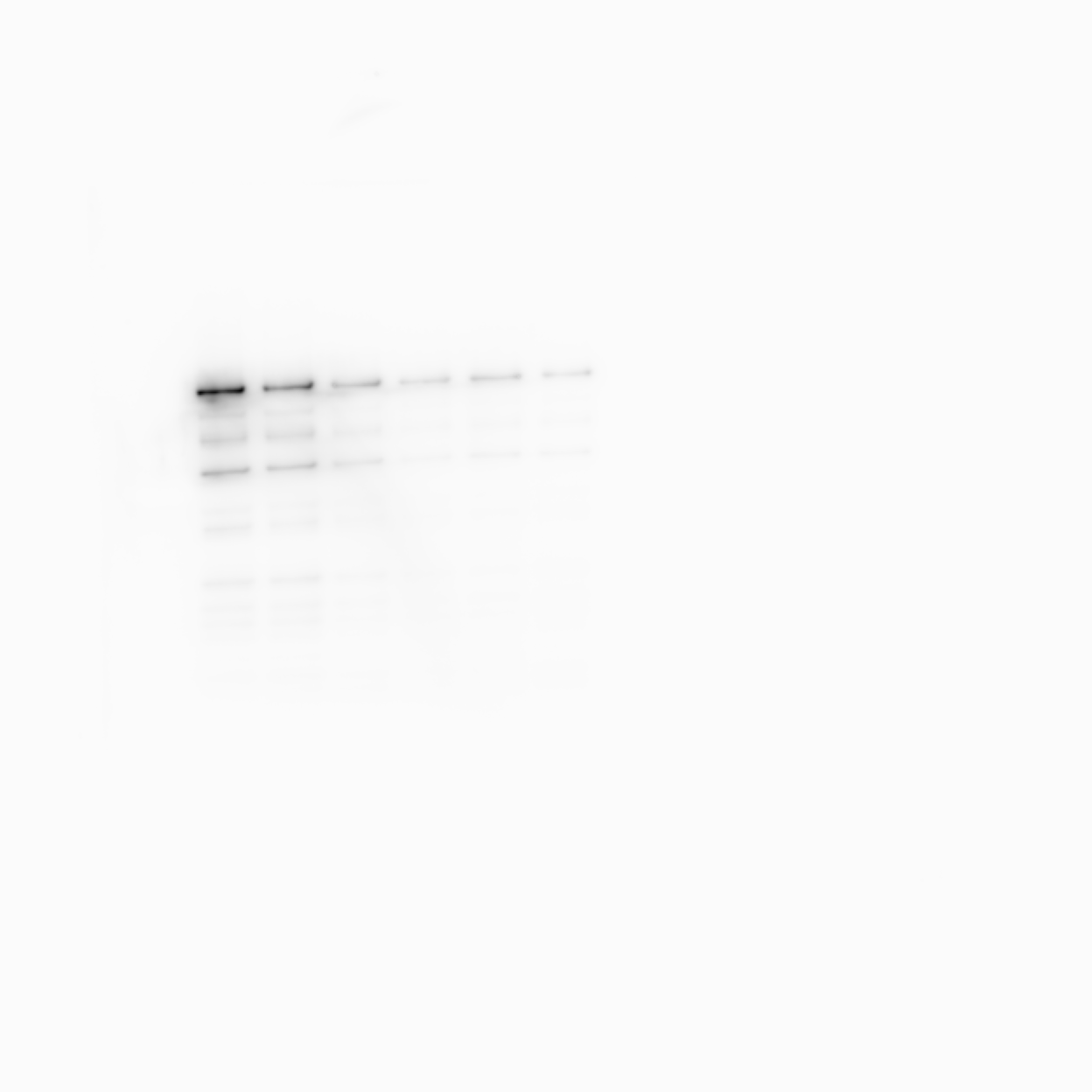

Supplement: Source data 1. [file elife-71662-supp1.zip › Figure 9ΓÇôsource data 6.tif]

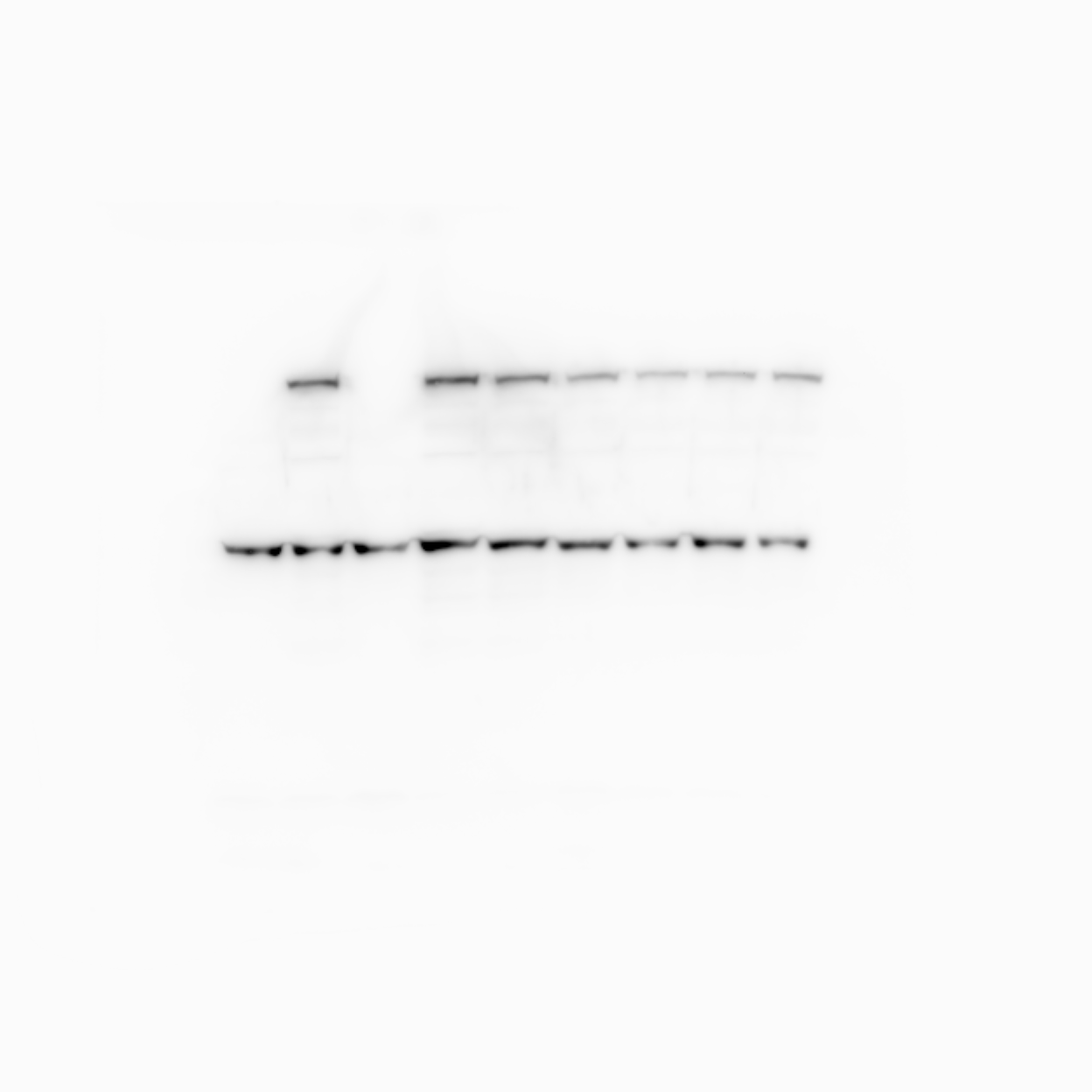

Supplement: Source data 1. [file elife-71662-supp1.zip › Figure 9ΓÇôsource data 7.tif]
